# Development of a novel sequence based real-time PCR assay for specific and sensitive detection of *Burkholderia pseudomallei* in clinical and environmental matrices

**DOI:** 10.1186/s12941-024-00693-4

**Published:** 2024-04-10

**Authors:** Pranjal Kumar Yadav, Suchetna Singh, Moumita Paul, Sanjay Kumar, S. Ponmariappan, Duraipandian Thavaselvam

**Affiliations:** 1 Biodetector Development Test and Evaluation Division, Defence Research & Development Establishment, Defence Research and Development Organization, Jhansi Road, Gwalior, Madhya Pradesh 474 002 India; 2 O/o DGLS, Defence Research and Development Organization, Ministry of Defence, SSPL Campus, Timarpur, New Delhi, 110 054 India

**Keywords:** Melioidosis, *Burkholderia pseudomallei*, *BPSS0664*, Real-time qPCR, Assay S664

## Abstract

**Background:**

Melioidosis, caused by the category B biothreat agent *Burkholderia pseudomallei*, is a disease with a high mortality rate and requires an immediate culture-independent diagnosis for effective disease management. In this study, we developed a highly sensitive qPCR assay for specific detection of *Burkholderia pseudomallei* and melioidosis disease diagnosis based on a novel target sequence.

**Methods:**

An extensive *in-silico* analysis was done to identify a novel and highly conserved sequence for developing a qPCR assay. The specificity of the developed assay was analyzed with 65 different bacterial cultures, and the analytical sensitivity of the assay was determined with the purified genomic DNA of *B. pseudomallei*. The applicability of the assay for *B. pseudomallei* detection in clinical and environmental matrices was evaluated by spiking *B. pseudomallei* cells in the blood, urine, soil, and water along with suitable internal controls.

**Results:**

A novel 85-nucleotide-long sequence was identified using *in-silico* tools and employed for the development of the highly sensitive and specific quantitative real-time PCR assay S664. The assay S664 was found to be highly specific when evaluated with 65 different bacterial cultures related and non-related to *B. pseudomallei*. The assay was found to be highly sensitive, with a detection limit of 3 *B. pseudomallei* genome equivalent copies per qPCR reaction. The detection limit in clinical matrices was found to be 5 × 10^2^ CFU/mL for both human blood and urine. In environmental matrices, the detection limit was found to be 5 × 10^1^ CFU/mL of river water and 2 × 10^3^ CFU/gm of paddy field soil.

**Conclusions:**

The findings of the present study suggest that the developed assay S664 along with suitable internal controls has a huge diagnostic potential and can be successfully employed for specific, sensitive, and rapid molecular detection of *B. pseudomallei* in various clinical and environmental matrices.

**Supplementary Information:**

The online version contains supplementary material available at 10.1186/s12941-024-00693-4.

## Introduction

*Burkholderia pseudomallei* is a category B biothreat agent responsible for causing melioidosis, a serious invasive disease of humans with a high case fatality rate [1]. *B. pseudomallei* is a saprophytic environmental pathogen predominantly found in rhizospheric soil, paddy fields, and standing streams [2]. The human population living in rural areas is at high risk of acquiring this infection mostly in the rainy season through direct contact with contaminated soil and surface water [3]. The most common symptoms of melioidosis are associated with respiratory and cardiovascular systems and its non-specific clinical presentation hampers diagnosis and also delays early treatment which characteristically leads to high fatality rate [4].

Currently, the culturing method used for the isolation and identification of bacteria from clinical and environmental samples is the gold standard for the diagnosis and detection of *B. pseudomallei*. However, it requires expertise, special selective media, and a long incubation period (4–5 days) which delays diagnosis. Furthermore, isolated cultures are usually misidentified as *Bacillus* or *Pseudomonas* species [5]. Serological tests such as Indirect Haemagglutination Assay, Enzyme-Linked Immunosorbent Assay, and Lateral Flow Immuno-assays are used for the detection of *B. pseudomallei*-specific antibodies. These methods are not reliable for accurate disease diagnosis, especially in highly endemic areas where a high rate of background seropositivity in healthy populations is observed [6]. So, to conquer the established boundaries of microbiological and serological test methods, direct nucleic acid amplification-based specific molecular detection methods have been developed. Real-time quantitative polymerase chain reaction (qPCR) assays have been identified to have high degrees of specificity and sensitivity for organism detection in the samples [7]. At present, the most promising assay for the detection of *B. pseudomallei* generally targets gene clusters of type III secretion system (T3SS). The *orf*2 within the T3SS1 is considered the gold standard for molecular identification of *B. pseudomallei* [8]. The other gene targets such as 16S rRNA, TTSS1*-orf*11, *mpr*A, *YLF*/*BTFC*, *BPSL1664*, *pha*C, *lpx*O, and Bp loci 8653 and 9438 have also been evaluated so far for their efficiency in the identification of *B. pseudomallei* [8, 9]. However, all the above-mentioned assays lack internal controls to monitor proper nucleic acid extraction and adequate nucleic acid amplification.

The genomic heterogeneity and high rate of genetic recombination are the few most striking features of *B. pseudomallei* [10]. The natural competency of *B. pseudomallei* for DNA uptake and catabolism adds to its genetic diversity [11]. Furthermore, efficient and simple gears have been developed for the compliant genetic manipulations in the genome of this category B biothreat agent [12]. Misuse of genetically manipulated or naturally occurring *B. pseudomallei* strains that lacks the specific target sequence will pose a serious threat to human life due to their high lethality. Hence, the existing assays are insufficient to counter and detect the altered pathogen in case of public health and biothreat emergencies. Therefore, there is an ever-increasing need to identify novel targets for specific detection and identification of *B. pseudomallei* in clinical and environmental settings. Keeping the above features of *B. pseudomallei* in mind, the present study was focused to develop a multiplex hydrolysis probe-based real-time qPCR assay targeting *in-silico* identified novel gene target. To the best of our knowledge, this is the first report describing the development of a novel multiplex qPCR assay employing suitable internal controls for melioidosis disease diagnosis and detection of *B. pseudomallei* in different environmental matrices.

## Materials and methods

### *In-silico* identification of specific target and primer-probe designing

For the identification of *B. pseudomallei*-specific novel candidate sequence, the genomic regions of *B. pseudomallei *absent in the genome of *Burkholderia mallei *were initially shortlisted [13]. The basis of such an analysis was that the *B. mallei* evolved as a deletion clone of *B. pseudomallei* [13, 14]. The obtained gene sequences of these genomic regions were then analyzed *in-silico* to derive unanimously unique sequences of *B. pseudomallei*. The nucleotide BLAST (BLASTn, https://blast.ncbi.nlm.nih.gov/blast.cgi) was done for the shortlisted genes against the RefSeq Genome Database (refseq genomes) of *B. pseudomallei*. A novel gene was finally selected based on its specificity and higher *B. pseudomallei* strain coverage in comparison to *orf*2. The complete sequence of the novel gene from all the available strains was subsequently obtained and aligned employing ClustalW in MEGA X software in order to identify the highly conserved region within the gene. Further, the BLASTn server was used to retrieve the *orf*2 sequences from available strains, and strain-wise comparative analysis for both the sequences was performed. Three sets of the primers and respective probes for the identified gene segment were initially designed by the PrimerQuest tool (https://sg.idtdna.com/pages/tools/primerquest) and then individually screened, analyzed, and sorted using the BLASTn server to reduce the possibility of cross-reactivity with other organisms [15].

### Bacterial culture condition and DNA preparation

The cultures of *B. pseudomallei* were grown on Ashdown’s agar medium containing 4% Glycerol (Fisher Scientific, #CAS 56-81-5), 1% Tryptone Soya Broth (HiMedia, #M011), 0.5 mg/L Crystal Violet (HiMedia, #GRM961), 5 mg/L Neutral Red (HiMedia, #RM122), and 1.5% Bacteriological Agar (HiMedia, #GRM026) supplemented with 5 mg/L of Gentamicin (HiMedia, #RM461) at 37 °C for 48–72 h [16]. The other cultures used in the study were grown on Brain Heart Infusion (BHI) agar medium (HiMedia, #M211) at 37 °C for 16 to 48 h. Obtained single colonies were inoculated into BHI broth (HiMedia, #M210) for DNA extraction. The genomic DNA from bacterial cells was extracted using DNeasy Blood and Tissue kit (Qiagen, #69504) in accordance with the manufacturer’s protocol. The purity and quantity of DNA were measured using NanoDrop (Thermo). Isolated purified bacterial genomic DNA was aliquoted and stored at -20 °C till further use.

### Hydrolysis probe-based qPCR assay S664

The qPCR assay S664 was performed using GoTaq Probe qPCR master mix (Promega, #A6102) on StepOne Real-Time PCR System (Applied Biosystems) and CFX96 Touch Real-Time PCR detection systems (Bio-Rad). The qPCR reactions were prepared in a total volume of 20 µL containing 1× master mix, 1000 nM of each forward and reverse primer, 250 nM hydrolysis probe (Eurofins genomics) and 2 µL of DNA. The list of primer and probes used in the study is mentioned in Table 1. The thermal profile of the assay consisted of 10 min of initial denaturation and polymerase activation at 95 °C followed by 40 cycles of denaturation at 95 °C for 15 s and annealing/extension at 60 °C for 60 s.

**Table 1:** Primers and probes used in the development of singleplex and multiplex qPCR assay S664 for detection of *B. pseudomallei* in clinical and environmental matrices

Primer/Probe	Sequence (5’→3’)	Purpose	Product length	Source
S664-F	GTAATTGTGACGGTCCTATCGTAATG	qPCR target	85 bp	This study
S664-R	TTTCATCCCAATAAATGTAGTCGTC			
S664-PB	FAM-ACGAATGCCTTGCCTTGTCCTCC-BHQ1			
*RNaseP*-F	AGATTTGGACCTGCGAGCG	Internal control (blood and urine)	65 bp	[18]
*RNaseP*-R	GAGCGGCTGTCTCCACAAGT			
*RNaseP*-PB	JOE-TTCTGACCTGAAGGCTCTGCGCG-BHQ1			
*cry1*-F	AGTTCGTGTCTGTCCGGGTC	Internal control (soil and water)	85 bp	[20]
*cry1*-R	CATGAATGGTTACGCAACCTTCT			
*cry1*-PB	Texas Red-ATCCCTCCTTGTACGCTGTGACACGAAGGA-BHQ2			

*S664 BPSS0664* response regulator protein gene, *RNaseP* ribonuclease P gene, *cry1* insecticidal crystal protein gene, *F* forward primer, *R* reverse primer, *PB* fluorescent labelled probe, *FAM* 6-carboxyfluorescein, *JOE* 4-5-dichlorodimethoxyfluorescein, *Texas Red* sulforhodamine 101 acid chloride, *BHQ1* & *BHQ2* black hole quencher 1 and 2

### Analytical sensitivity and specificity of assay S664

To determine the analytical sensitivity of assay S664, a 10-fold serially diluted *B. pseudomallei* genomic DNA ranging from 3 × 10^6 ^to 3 × 10^− 1 ^genome equivalent (GE) copies/reaction was used. The amount of DNA was converted to GE copies based on the size of *B. pseudomallei* genome (7.25 × 10^6 ^bp) [13, 16]. All the qPCRs were carried out in triplicate, and at least two separate experiments were performed. The obtained cycle threshold (Ct) values were used to generate standard curve. The efficiency of the assay was calculated by the formula E = (-1 + 10^− 1/slope^) × 100 using the slope of the standard curve [17]. The coefficient of variation for inter-assay and intra-assay were calculated. The specificity of the developed assay S664 was tested three times by incorporating genomic DNA (∼ 10 ng) from *B. pseudomallei-*related and non-related bacterial cultures (Table 2). *B. pseudomallei* (NCTC 13392) served as a positive control and nuclease-free water served as no template control (NTC) in the specificity analysis.

**Table 2:** Bacterial cultures used in the study to determine the specificity of developed qPCR assay S664

Organism (*n* = 65)		Source	No. of isolates/strains tested (sample type)	qPCR result
*Burkholderia pseudomallei* (*n* = 17)				
	Standard strains	NCTC 13392, NCTC 6700, NCTC 4845, NCTC 10274	4 (purified DNA)	Positive (4)
	Clinical isolates	Clinical isolates	9 (purified DNA)	Positive (9)
	Soil isolates	Soil isolates	4 (purified DNA)	Positive (4)
*Burkholderia/Delftia/Ralstonia* (*n* = 9)				
	*Burkholderia thailandensis*	Clinical isolate	1 (purified DNA)	Negative (1)
	*Burkholderia mallei*	NCTC 10245	1 (purified DNA)	Negative (1)
	*Burkholderia cepacia*	MTCC 1617, MTCC 438	2 (purified DNA)	Negative (2)
	*Burkholderia gladioli*	MTCC 1888	1 (purified DNA)	Negative (1)
	*Delftia acidovorans*	MTCC 104	1 (purified DNA)	Negative (1)
	*Ralstonia eutropha*	MTCC 1285	1 (purified DNA)	Negative (1)
	*Ralstonia insidiosa*	ATCC 49129	1 (purified DNA)	Negative (1)
	*Ralstonia pickettii*	MTCC 648	1 (purified DNA)	Negative (1)
Biothreat agents/simulants (*n* = 9)				
	*Bacillus anthracis*	Clinical isolate	2 (purified DNA)	Negative (2)
	*Bacillus globigii*	ATCC 9372	1 (purified DNA)	Negative (1)
	*Brucella abortus*	NCTC 11363	1 (purified DNA)	Negative (1)
	*Brucella canis*	NCTC 11365	1 (purified DNA)	Negative (1)
	*Brucella melitensis*	NCTC 10094	1 (purified DNA)	Negative (1)
	*Coxiella burnetii*	Nine mile I	1 (purified DNA)	Negative (1)
	*Francisella tularensis* LVS	NCTC 10857	1 (purified DNA)	Negative (1)
	*Pantoea agglomerans*	ATCC 33243	1 (purified DNA)	Negative (1)
Bacteria of clinical relevance (*n* = 15)				
	*Brevundimonas diminuta*	ATCC 11568	1 (purified DNA)	Negative (1)
	*Corynebacterium pseudotuberculosis*	MTCC 3158	1 (purified DNA)	Negative (1)
	*Escherichia coli*	ATCC 35218	1 (purified DNA)	Negative (1)
	*Klebsiella pneumoniae*	ATCC 27736	1 (purified DNA)	Negative (1)
	*Ochrobactrum oryzae*	MTCC 4195	1 (purified DNA)	Negative (1)
	*Pasteurella multocida*	MTCC 1148	1 (purified DNA)	Negative (1)
	*Pasteurella pneumotropica*	MTCC 656	1 (purified DNA)	Negative (1)
	*Proteus vulgaris*	ATCC 6380P	1 (purified DNA)	Negative (1)
	*Pseudomonas aeruginosa*	ATCC 15442	1 (purified DNA)	Negative (1)
	*Pseudomonas citronellolis*	MTCC 1191	1 (purified DNA)	Negative (1)
	*Pseudomonas putida*	MTCC 102	1 (purified DNA)	Negative (1)
	*Salmonella typhi*	Lab culture	1 (purified DNA)	Negative (1)
	*Shigella dysenteriae*	Lab culture	1 (purified DNA)	Negative (1)
	*Staphylococcus aureus*	ATCC 11632	1 (purified DNA)	Negative (1)
	*Yersinia enterocolitica*	ATCC 55075	1 (purified DNA)	Negative (1)
Plant pathogen/symbionts (*n* = 5)				
	*Pseudomonas syringae*	MTCC 1604	1 (purified DNA)	Negative (1)
	*Rhizobium meliloti*	MTCC 3402	1 (purified DNA)	Negative (1)
	*Rhizobium radiobacter*	MTCC 6702	1 (purified DNA)	Negative (1)
	*Rhizobium rhizogenes*	MTCC 2364	1 (purified DNA)	Negative (1)
	*Rhizobium trifoli*	MTCC 905	1 (purified DNA)	Negative (1)
Other bacteria (*n* = 10)				
	*Bacillus mycoides*	MTCC 7538	1 (purified DNA)	Negative (1)
	*Bacillus thuringiensis*	NCIM 5112, MTCC 868, MTCC 869	3 (purified DNA)	Negative (3)
	*Corynebacterium ammoniagenes*	MTCC 1816	1 (purified DNA)	Negative (1)
	*Corynebacterium callunae*	MTCC 700	1 (purified DNA)	Negative (1)
	*Corynebacterium glutamicum*	MTCC 26	1 (purified DNA)	Negative (1)
	*Microbulbifer elongatus*	MTCC 2426	1 (purified DNA)	Negative (1)
	*Pseudomonas fragi*	MTCC 510	1 (purified DNA)	Negative (1)
	*Vibrio fischeri*	MTCC 1738	1 (purified DNA)	Negative (1)

*NCTC* National Collection of Type Culture, *ATCC* American Type Culture Collection, *MTCC* Microbial Type Culture Collection and Gene Bank, *NCIM* National Collection of Industrial Microorganisms

### Detection of *B. pseudomallei* in clinical matrices

For the feasibility of assay S664 to detect *B. pseudomallei* in clinical samples, a multiplex assay S664 was developed using the novel target *BPSS0664* and the human *RNase*P gene as endogenous control (Table 1) [18]. The multiplex qPCR reaction was prepared in total 20 µL volume containing 1× master mix, 250 nM of forward primer, reverse primer, and probe of gene *BPSS0664*, 750 nM of forward and reverse primer, and 250 nM hydrolysis probe of gene *RNase*P and 2 µL of DNA. To determine the limit of detection in clinical matrices, 10-fold serial dilutions of *B. pseudomallei* cells were spiked in healthy human blood collected in EDTA-coated vials [19] and urine ranging from 5 × 10^7^ to 5 × 10^0^ cells/mL. The total DNA was extracted from spiked blood and urine using DNeasy Blood and Tissue kit according to the manufacturer’s instructions. The qPCR was performed in triplicates for each dilution along with non-spiked control and NTC to determine the detection limit.

### Detection of *B. pseudomallei* in environmental matrices

*B. pseudomallei* prefer moist, nutrient-rich rhizospheric soil and also found in water bodies [2]. For the applicability of the developed assay S664 to detect *B. pseudomallei* in water and soil, a multiplex assay S664 has been developed using the novel target *BPSS0664* and the *cry*1 gene of *Bacillus thuringiensis* (Table 1) [20]. The multiplex qPCR reaction was prepared in total 20 µL volume containing 1× master mix, 250 nM of forward primer, reverse primer, and probe of gene *BPSS0664*, 750 nM of forward and reverse primer and 250 nM of hydrolysis probe of gene *cry*1 and 2 µL of DNA. The river water was collected from the Narmada River, Khandwa, Madhya Pradesh, India (GPS coordinates: N 22° 14’ 36.58”, E 76° 9’ 39.79”) and paddy field soil from Bharatpur village in Lucknow, Uttar Pradesh, India (GPS coordinates: N 27° 2’ 43.05”, E 80° 53’ 39.74”) [16]. The river water was spiked with *B. pseudomallei* cells at a concentration of 5 × 10^7^ to 5 × 10^0^ CFU/mL of water. The *B. thuringiensis* cells (10^5^) were chosen as an internal control for DNA extraction and PCR amplification for detecting *B. pseudomallei* in water samples [21]. The concentration of *B. thuringiensis* cells was empirically determined to yield cycle threshold (Ct) values between 28 and 30 along with *B. pseudomallei*-specific amplification. The DNA was extracted from spiked water (*B. pseudomallei* and *B. thuringiensis* cells) using the DNeasy Blood and Tissue kit according to the manufacturer’s instructions. The paddy field soil was spiked with *B. pseudomallei* cells at a concentration of 2 × 10^7^ to 2 × 10^0^ CFU/gm of soil. The *B. thuringiensis* spores (10^5^) were chosen as an internal control for DNA extraction and PCR amplification for detecting *B. pseudomallei* in soil samples [20]. The concentration of *B. thuringiensis* spores was empirically determined to yield Ct values between 28 and 30 along with *B. pseudomallei*-specific amplification. The total DNA from spiked soil (*B. pseudomallei* and *B. thuringiensis* cells) was extracted using the NucleoSpin Soil kit (Macherey-Nagel, #REF740780.50) according to the manufacturer’s instructions. All the qPCR reactions for spiked water as well as spiked soil were performed in triplicates for each dilution along with the non-spiked control and NTC to determine the detection limit.

## Results

### Identification of *B. pseudomallei*-*s*pecific target and primer-probe designing

The results of the *in-silico* studies showed that the 85-bp region within the gene *BPSS0664* was unique and had no significant similarity with the sequences of related or non-related organisms. The presence of *in-silico* identified novel gene sequence in 1794 out of 1796 strains of *B. pseudomallei* indicates enhanced strain coverage in contrast to *orf*2 of T3SS1 which is present in only 1791 strains. *BPSS0664* is exclusively present in five strains (1258a, NRF80Bp1, SBCT-RF80-BP1, NAU14B-9, and MSHR1879) that are devoid of *orf*2 sequence (Table S1). Multiple sequence alignment analysis revealed that the 85-bp region of the *BPSS0664* gene was highly conserved (Fig. S1). The forward and reverse primers amplifying an 85-bp long amplicon along with a labelled hydrolysis probe were designed and used for the real-time PCR assay S664 development (Table 1).

### Analytical sensitivity and specificity of the assay S664

**Fig. 1:**
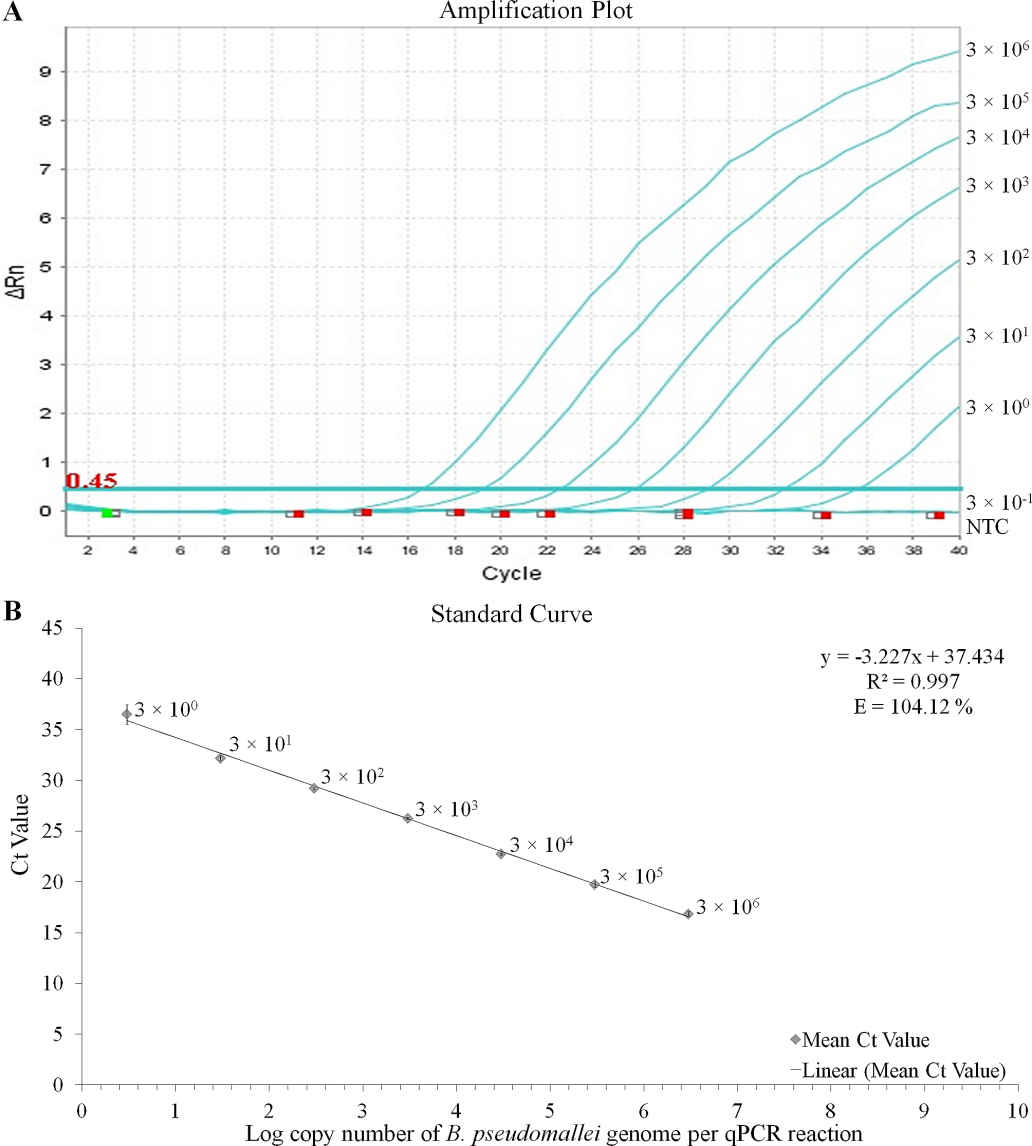
Analytical sensitivity of the assay S664 (**A**) Amplification plot showing sensitivity of 10-fold serially diluted *B. pseudomallei* GE copies from 3 × 10^6^ to 3 × 10^0^ per qPCR reaction (**B**) Graph plot showing straight calibration line for 10-fold serially diluted log *B. pseudomallei* GE copies from 3 × 10^6^ to 3 × 10^0^ per qPCR reaction

The limit of detection of the developed novel sequence-based assay S664 was found to be 3 GE copies of *B. pseudomallei* genome per qPCR reaction (Fig. 1A). A linear calibration line was obtained in the standard curve plotted using mean Ct values against the log concentration of a 10-fold serially diluted *B. pseudomallei* genomic DNA with a linear model equation of y = -3.227x + 37.434. A strong linear inverse relationship was observed between log_10_GE copies of *B. pseudomallei *and Ct values with a linear regression coefficient value (R^2^) of 0.997. The efficiency of assay S664 was found to be 104.12% (Fig. 1B). The intra-assay variations were estimated between 0.4% and 2.7% while inter-assay variations were between 0.3% and 1.9%. The developed assay S664 has specifically detected standard strains, soil isolates, and clinical isolates of *B. pseudomallei*, and no cross-reactivity was observed with bacterial species within the genus *Burkholderia* or other closely related organisms. Moreover, no cross-reactivity was also observed with other non *B. pseudomallei* related organisms (Table 2).

### The feasibility of assay S664 to detect *B. pseudomallei* in clinical samples

**Fig. 2:**
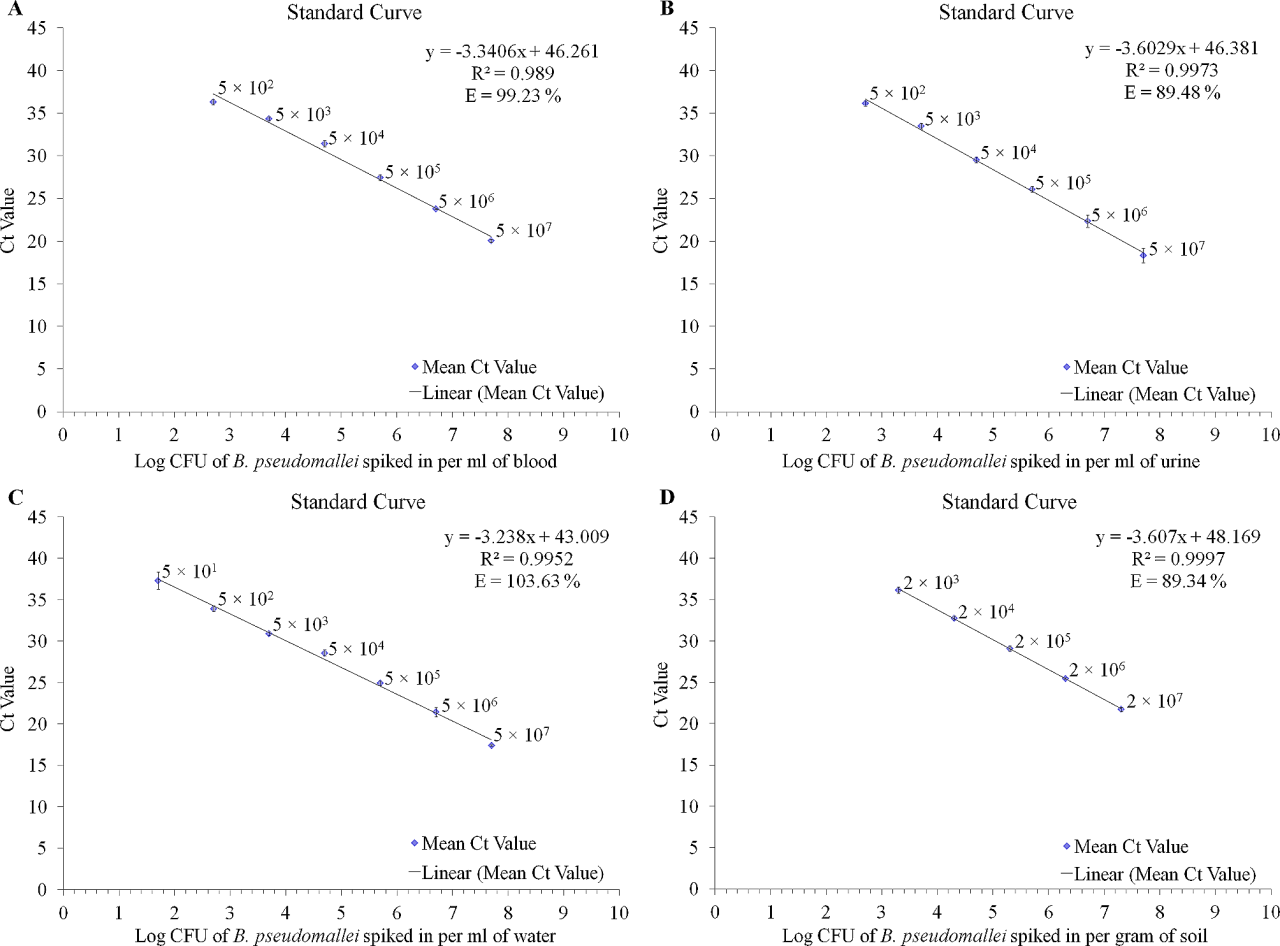
The feasibility of multiplex assay S664 for detection of *B. pseudomallei *in clinical and environmental matrices (**A**) Graph plot showing straight calibration line for detection of *B. pseudomallei* cells spiked in human blood from 5 × 10^7^ to 5 × 10^2^ CFU/mL (**B**) Graph plot showing straight calibration line for detection of *B. pseudomallei* cells spiked in human urine from 5 × 10^7^ to 5 × 10^2^ CFU/mL (**C**) Graph plot showing straight calibration line for detection of *B. pseudomallei* cells spiked in river water from 5 × 10^7^ to 5 × 10^1^ CFU/mL (**D**) Graph plot showing straight calibration line for detection of *B. pseudomallei* cells spiked in paddy field soil in concentration of 2 × 10^7^ to 2 × 10^3^ CFU/gm of soil

The feasibility of the developed multiplex assay S664 for the clinical diagnosis of melioidosis was assessed by spiking healthy human blood and urine with *B. pseudomallei* cells and employing suitable internal control (*RNase*P). The developed multiplex assay S664 was found to be highly sensitive with a detection limit of 5 × 10^2^ CFU/mL for both human blood and urine. The efficiency of multiplex assay S664 was found to be 99.2% and 89.5% for the detection of *B. pseudomallei* in human blood and urine respectively (Fig. 2A and B). The *RNase*P gene used as a control for nucleic acid extraction and amplification was readily detected in all the spiked clinical samples with a mean Ct value (± SD) of 23.9 ± 0.8 and 29.0 ± 0.8 for human blood and urine respectively.

### The feasibility of assay S664 to detect *B. pseudomallei* in environmental samples

The feasibility of the developed multiplex assay S664 in detecting *B. pseudomallei* from the environmental matrices namely, water and soil was assessed by spiking *B. pseudomallei* cells along with suitable internal controls. The detection limit of assay S664 in *B. pseudomallei*-spiked river water was found to be 5 × 10^1^ CFU/mL and no amplification was observed in non-spiked control river water (Fig. 2C). The *B. thuringiensis* vegetative cells used as a control for nucleic acid extraction and amplification was readily detected in all spiked river water samples with a mean Ct value of 29.9 ± 1.0. The detection limit of assay S664 in spiked paddy field soil was found to be 2 × 10^3^ CFU/gm of soil and no amplification for *B. pseudomallei* was observed in non-spiked control soil (Fig. 2D). The *B. thuringiensis* spores used as a control for nucleic acid extraction and amplification was readily detected in all spiked paddy field soil samples with a mean Ct value of 28.3 ± 1.1. The efficiency of the multiplex assay S664 was 103.6% and 89.3% in *B. pseudomallei*-spiked river water and paddy field soil respectively.

## Discussion

*B. pseudomallei* is an emerging pathogen as well as a potential biothreat agent owing to its remarkable capability to survive in extreme environmental conditions [22–25]. The disease melioidosis is acquired through direct contact with a pathogen from a contaminated environment [4]. The non-specific clinical manifestation leads to an inaccurate diagnosis on clinical grounds. The special culture method, which is a mainstay for diagnosis is confounded by its slow growth, requirement of special selective media, and expertise in identifying *B. pseudomallei* culture. All these limiting factors are collectively accountable for the high case fatality rate [6]. Therefore, specific and rapid identification of the pathogen is essential for the early control and prevention of melioidosis. Molecular detection techniques such as PCRs and other isothermal assays offer several advantages over conventional serological methods in terms of sensitivity and specificity. The reported molecular assays are mostly based on *orf*2 of T3SS1 of *B. pseudomallei* [26–29]. O*rf*2 is available in 1791 genomic assemblies of *B. pseudomallei* out of 1796. Hence, the assays based on *orf*2 can detect only 1791 strains of *B. pseudomallei* out of 1796 due to the lost target sequence. Furthermore, the highly plastic genome of *B. pseudomallei* is exceptionally vulnerable to natural genetic recombination and artificial genetic manipulations which can alter the outcome of molecular assays based on the *orf*2 sequence [10–13]. Additionally, the molecular assays developed in the past lacked suitable internal controls for appropriate monitoring of nucleic acid extraction and amplification from the clinical and environmental samples [9]. Therefore, there is an indispensable need to develop molecular assays based on novel gene targets accompanied by internal controls for the specific, sensitive, and reliable detection of *B. pseudomallei* in clinical and environmental samples.

In the present study, we have identified a novel and highly specific 85-bp-long nucleotide sequence within the *BPSS0664* gene using extensive bioinformatic analysis. The identified sequence is highly conserved in the genomes of 1794 *B. pseudomallei *strains out of 1796. The comparative analysis of *orf*2 and *BPSS0664* suggests the presence of both targets in 1789 strains of *B. pseudomallei*, whereas *BPSS0664* is exclusively present in 5 strains i.e. 1258a (human isolate, Thailand), NRF80Bp1 (environmental isolate, Thailand), SBCT-RF80-BP1 (environmental isolate, Thailand), NAU14B-9 (environmental isolate, Australia), and MSHR1879 (human isolate, Australia) which are lacking the *orf*2 sequence and hence, the *in-silico* identified novel gene *BPSS0664* has an advantage over *orf*2 for specific and sensitive assay development. This newly identified gene sequence was used for the development of the hydrolysis probe-based qPCR assay S664. The analytical sensitivity of the developed qPCR assay S664 was evaluated with freshly isolated genomic DNA of *B. pseudomallei* (NCTC 13392). The assay S664 could detect 3 GE copies of the  genome per reaction which is more sensitive than reported real-time PCR assays [16, 29–32]. The specificity of assay S664 was further evaluated with 65 different *B. pseudomallei*-related and non-related bacterial cultures. The assay S664 was found to be highly specific for the identification of *B. pseudomallei* as no cross-reactivity was observed with other species of the genus *Burkholderia* (*B. thailandensis, B. mallei, B. cepacia*, and *B. gladioli*). Further, no cross-reactivity of the newly developed assay was also observed with related bacterial pathogens classified in group proteobacteria including *Brucella*, *Coxiella*, *Francisella*, *Pseudomonas*, *Klebsiella*, *Salmonella* and *Shigella* as well as other bacteria used in the present study.

The diagnostic and detection applicability of the assay S664 to detect *B. pseudomallei* in clinical and environmental samples, respectively, was evaluated by spiking *B. pseudomallei* cells in human blood, urine, river water, and paddy field soil. To ensure the proper nucleic acid extraction from different clinical and environmental matrices and to differentiate a true from a false negative result, the singleplex assay S664 was translated into a multiplex assay by incorporating suitable internal controls. For the clinical diagnosis of melioidosis in humans, a multiplex assay incorporating *BPSS0664* and the *RNase*P gene as an extraction and amplification control was developed. The basis for the selection of the *RNase*P is its presence in every human cell, and hence it can be readily detectable in all human clinical samples [33]. The developed multiplex assay S664 was found to be highly sensitive in the detection of *B. pseudomallei* in clinical matrices with a detection limit of 5 × 10^2^ CFU/mL for both human blood and urine. The assay S664 has higher sensitivity as compared to *orf*2-based real-time qPCR assay in clinical matrices [29, 32, 34]. The amplification of the *RNase*P gene used as an internal control was also observed in all the *B. pseudomallei* spiked and non-spiked human blood and urine samples and no cross-reactivity was observed with human DNA. The lower assay efficiency in human urine (89.5%) was observed as compared to human blood (99.2%), which could be due to the presence of PCR inhibitors in urine samples such as urea [35, 36]. Together with the application of *RNase*P as the internal control for both nucleic acid extraction and amplification, the developed multiplex assay assures a highly reliable and specific diagnosis of melioidosis in human clinical samples.

For the detection of *B. pseudomallei* in environmental samples such as water and soil which are the primary sources of infection, a multiplex assay incorporating *BPSS0664* and the *cry*1 gene as an extraction and amplification control was developed [20]. The *B. thuringiensis* vegetative cells and spores were spiked to water and soil samples, respectively, before the nucleic acid extraction. The multiplex assay S664 could detect 5 × 10^1 ^cells of *B. pseudomallei* per mL of water and 2 × 10^3 ^cells of *B. pseudomallei* per gm of soil which is higher than the detection limit reported by Saxena et al. [37] and similar to the detection limit reported by Peng et al. [32]. The amplification of the *cry*1 gene was observed in all *B. thuringiensis* spiked water and soil samples with Ct values ranging from 28 to 31. The lower assay efficiency in paddy field soil (89.3%) was observed as compared to the river water (103.6%) which could be due to the presence of PCR inhibitors in soil samples such as humic substances [36, 38]. Moreover, no amplification was observed with total DNA isolated from unspiked water and soil which are the primary habitats of many micro and macroorganisms showing the high degree of specificity of developed multiplex assay S664 [39, 40]. These results indicate the potential usefulness of the developed multiplex assay using the *cry*1 gene as an internal control for the detection of *B. pseudomallei* in environmental samples.

In conclusion, the developed multiplex qPCR assay targeting a novel gene with suitable internal controls has the potential for both sensitive and specific melioidosis disease diagnosis and it can provide an early and specific detection of *B. pseudomallei* in environmental samples in an outbreak or in a biothreat scenario. Altogether, the novel assay S664 can be a potential substitute for *orf*2-based molecular assays for detecting *B. pseudomallei* in diverse clinical and environmental matrices.

### Electronic supplementary material

Below is the link to the electronic supplementary material.

Supplementary Material 1

Supplementary Material 2

## Data Availability

All data generated or analysed during this study are included in this article [and its supplementary information files].
