# Supplementary figures and images for: Development of a novel sequence based real-time PCR assay for specific and sensitive detection of Burkholderia pseudomallei in clinical and environmental matrices
Source: Ann Clin Microbiol Antimicrob. 2024 Apr 10;23:30. doi: 10.1186/s12941-024-00693-4 (PMC11007888; doi:10.1186/s12941-024-00693-4)

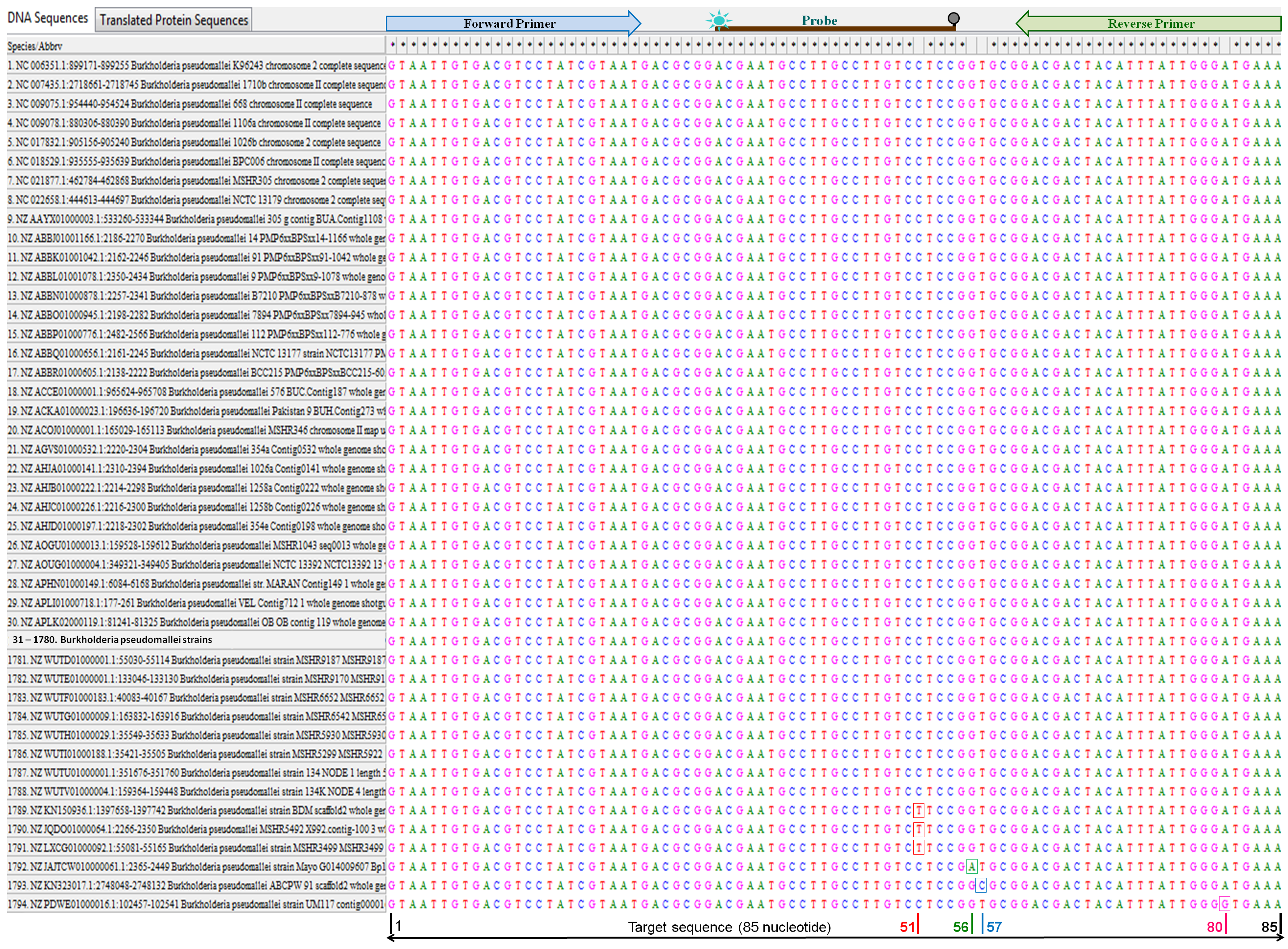

Supplement: Supplementary file 1 — Supplementary Material 1 [file 12941_2024_693_MOESM1_ESM.tif]
