# Supplementary material for: Development of a novel sequence based real-time PCR assay for specific and sensitive detection of Burkholderia pseudomallei in clinical and environmental matrices
Source: Ann Clin Microbiol Antimicrob. 2024 Apr 10;23:30. doi: 10.1186/s12941-024-00693-4 (PMC11007888; doi:10.1186/s12941-024-00693-4)
Supplement: Supplementary file 2 — Supplementary Material 2 [file 12941_2024_693_MOESM2_ESM.docx]

**Supplementary table S1** Comparative table for presence of identified novel target *BPSS0664* and *orf2* of T3SS1 in different *B. pseudomallei* strains

| S. No. | *B. pseudomallei* strains  (n = 1796) | *BPSS0664*  (n=1794) | *orf2*  (n=1791) |
| --- | --- | --- | --- |
|  | *B. pseudomallei* K96243 (NC_006351) | Present | Present |
|  | *B. pseudomallei* 1710b | Present | Present |
|  | *B. pseudomallei* 668 | Present | Present |
|  | *B. pseudomallei* 1106a | Present | Present |
|  | *B. pseudomallei* 1026b | Present | Present |
|  | *B. pseudomallei* BPC006 | Present | Present |
|  | *B. pseudomallei* MSHR305 | Present | Present |
|  | *B. pseudomallei* NCTC 13179 | Present | Present |
|  | *B. pseudomallei* 305 g | Present | Present |
|  | *B. pseudomallei* 14 PMP6xxBPSxx14 | Present | Present |
|  | *B. pseudomallei* 91 PMP6xxBPSxx91 | Present | Present |
|  | *B. pseudomallei* 9 PMP6xxBPSxx9 | Present | Present |
|  | *B. pseudomallei* B7210 PMP6xxBPSxxB7210 | Present | Present |
|  | *B. pseudomallei* 7894 PMP6xxBPSxx7894 | Present | Present |
|  | *B. pseudomallei* 112 PMP6xxBPSxx112 | Present | Present |
|  | *B. pseudomallei* NCTC 13177 | Present | Present |
|  | *B. pseudomallei* BCC215 PMP6xxBPSxxBCC215 | Present | Present |
|  | *B. pseudomallei* 576 BUC | Present | Present |
|  | *B. pseudomallei* Pakistan 9 BUH | Present | Present |
|  | *B. pseudomallei* MSHR346 | Present | Present |
|  | *B. pseudomallei* 354a | Present | Present |
|  | *B. pseudomallei* 1026a | Present | Present |
|  | *B. pseudomallei* 1258a | Present | **Absent** |
|  | *B. pseudomallei* 1258b | Present | Present |
|  | *B. pseudomallei* 354e | Present | Present |
|  | *B. pseudomallei* MSHR1043 | Present | Present |
|  | *B. pseudomallei* NCTC 13392 | Present | Present |
|  | *B. pseudomallei* str. MARAN | Present | Present |
|  | *B. pseudomallei* VEL | Present | Present |
|  | *B. pseudomallei* OB OB | Present | Present |
|  | *B. pseudomallei* OS OS | Present | Present |
|  | *B. pseudomallei* CB CB | Present | Present |
|  | *B. pseudomallei* CS CS | Present | Present |
|  | *B. pseudomallei* 406e BP 406E | Present | Present |
|  | *B. pseudomallei* 4900CFPatient1 | Present | Present |
|  | *B. pseudomallei* MSHR465a | Present | Present |
|  | *B. pseudomallei* Gu1909a | Present | Present |
|  | *B. pseudomallei* MSHR1950 | **Absent** | Present |
|  | *B. pseudomallei* NAU14B6 | Present | Present |
|  | *B. pseudomallei* NAU20B16 | Present | Present |
|  | *B. pseudomallei* NAU24B3 | Present | Present |
|  | *B. pseudomallei* NAU44A6 | Present | Present |
|  | *B. pseudomallei* NCTC 13178 | Present | Present |
|  | *B. pseudomallei* NCTC 13179 | Present | Present |
|  | *B. pseudomallei* PB08298010 | Present | Present |
|  | *B. pseudomallei* PHLS9 | Present | Present |
|  | *B. pseudomallei* RF67BP1 | Present | Present |
|  | *B. pseudomallei* RF6BP15 | Present | Present |
|  | *B. pseudomallei* NRF80Bp1 | Present | **Absent** |
|  | *B. pseudomallei* RF85Bp37 | Present | Present |
|  | *B. pseudomallei* RNS3Bp1 | Present | Present |
|  | *B. pseudomallei* RNS3Bp6 | Present | Present |
|  | *B. pseudomallei* Songkhla34W2 | Present | Present |
|  | *B. pseudomallei* MSHR338 | Present | Present |
|  | *B. pseudomallei* str. NCTC 13392 substr. morphotype 1 | Present | Present |
|  | *B. pseudomallei* str. NCTC 13392 substr. morphotype 2 | Present | Present |
|  | *B. pseudomallei* str. NCTC 13392 substr. morphotype 3 | Present | Present |
|  | *B. pseudomallei* str. NCTC 13392 substr. morphotype 5 | Present | Present |
|  | *B. pseudomallei* str. NCTC 13392 substr. morphotype 6 | Present | Present |
|  | *B. pseudomallei* str. NCTC 13392 substr. morphotype 7 | Present | Present |
|  | *B. pseudomallei* str. NCTC 13392 substr. morphotype 8 | Present | Present |
|  | *B. pseudomallei* str. NCTC 13392 substr. morphotype 9 | Present | Present |
|  | *B. pseudomallei* MSHR5848 | Present | Present |
|  | *B. pseudomallei* MSHR5858 | Present | Present |
|  | *B. pseudomallei* HBPUB10134a | Present | Present |
|  | *B. pseudomallei* HBPUB10303a | Present | Present |
|  | *B. pseudomallei* str. NCTC 13392 substr. morphotype 10 | Present | Present |
|  | *B. pseudomallei* MSHR6137 | Present | Present |
|  | *B. pseudomallei* strain WRAIR 286 | Present | Present |
|  | *B. pseudomallei* strain EM10266/01 | Present | Present |
|  | *B. pseudomallei* strain KAS | Present | Present |
|  | *B. pseudomallei* strain 15-10 | Present | Present |
|  | *B. pseudomallei* strain 20 | Present | Present |
|  | *B. pseudomallei* strain 54 | Present | Present |
|  | *B. pseudomallei* strain 2746b | Present | Present |
|  | *B. pseudomallei* strain 33 | Present | Present |
|  | *B. pseudomallei* strain 10 | Present | Present |
|  | *B. pseudomallei* strain AH1 | Present | Present |
|  | *B. pseudomallei* strain 4094-0081 | Present | Present |
|  | *B. pseudomallei* strain D10 7310-3154 | Present | Present |
|  | *B. pseudomallei* strain EB5661 | Present | Present |
|  | *B. pseudomallei* strain 4009-19 | Present | Present |
|  | *B. pseudomallei* strain 35 | Present | Present |
|  | *B. pseudomallei* strain 153 | Present | Present |
|  | *B. pseudomallei* strain 497/96 | Present | Present |
|  | *B. pseudomallei* strain 27/96 | Present | Present |
|  | *B. pseudomallei* strain D6 8346-3058 | Present | Present |
|  | *B. pseudomallei* strain EM1157 | Present | Present |
|  | *B. pseudomallei* strain 17/96 | Present | Present |
|  | *B. pseudomallei* strain SW1 | Present | Present |
|  | *B. pseudomallei* strain JAM | Present | Present |
|  | *B. pseudomallei* strain 490 | Present | Present |
|  | *B. pseudomallei* strain 109/96 | Present | Present |
|  | *B. pseudomallei* strain TRF661 | Present | Present |
|  | *B. pseudomallei* strain 79/96 | Present | Present |
|  | *B. pseudomallei* strain DB | Present | Present |
|  | *B. pseudomallei* strain 35/96 | Present | Present |
|  | *B. pseudomallei* strain C27 | Present | Present |
|  | *B. pseudomallei* strain C64 | Present | Present |
|  | *B. pseudomallei* strain C41 | Present | Present |
|  | *B. pseudomallei* strain C18 | Present | Present |
|  | *B. pseudomallei* strain C38 | Present | Present |
|  | *B. pseudomallei* strain C9 | Present | Present |
|  | *B. pseudomallei* strain C4 | Present | Present |
|  | *B. pseudomallei* strain C46 | Present | Present |
|  | *B. pseudomallei* strain C49 | Present | Present |
|  | *B. pseudomallei* strain C39 | Present | Present |
|  | *B. pseudomallei* strain C19 | Present | Present |
|  | *B. pseudomallei* strain C65 | Present | Present |
|  | *B. pseudomallei* strain C12 | Present | Present |
|  | *B. pseudomallei* strain C61 | Present | Present |
|  | *B. pseudomallei* strain C59 | Present | Present |
|  | *B. pseudomallei* strain 6 | Present | Present |
|  | *B. pseudomallei* strain EY8 | Present | Present |
|  | *B. pseudomallei* strain DM15342/00 | Present | Present |
|  | *B. pseudomallei* strain 612 | Present | Present |
|  | *B. pseudomallei* strain DB30729/00 | Present | Present |
|  | *B. pseudomallei* strain BRI | Present | Present |
|  | *B. pseudomallei* strain E8 | Present | Present |
|  | *B. pseudomallei* strain AH4 | Present | Present |
|  | *B. pseudomallei* strain I6 4043-3096 | Present | Present |
|  | *B. pseudomallei* strain C14 | Present | Present |
|  | *B. pseudomallei* strain 77/96 | Present | Present |
|  | *B. pseudomallei* strain C57 | Present | Present |
|  | *B. pseudomallei* strain C55 | Present | Present |
|  | *B. pseudomallei* strain C6 | Present | Present |
|  | *B. pseudomallei* strain C67 | Present | Present |
|  | *B. pseudomallei* strain 4 | Present | Present |
|  | *B. pseudomallei* strain EM2107 | Present | Present |
|  | *B. pseudomallei* strain Soil | Present | Present |
|  | *B. pseudomallei* strain EY9 | Present | Present |
|  | *B. pseudomallei* strain C34 | Present | Present |
|  | *B. pseudomallei* strain C23 | Present | Present |
|  | *B. pseudomallei* strain C20 | Present | Present |
|  | *B. pseudomallei* 1655 | Present | Present |
|  | *B. pseudomallei* 406e | Present | Present |
|  | *B. pseudomallei* Pasteur 52237 | Present | Present |
|  | *B. pseudomallei* S13 | Present | Present |
|  | *B. pseudomallei* strain AH3 | Present | Present |
|  | *B. pseudomallei* strain 59 | Present | Present |
|  | *B. pseudomallei* strain EY4 | Present | Present |
|  | *B. pseudomallei* strain DC | Present | Present |
|  | *B. pseudomallei* strain E0387 | Present | Present |
|  | *B. pseudomallei* strain 11 | Present | Present |
|  | *B. pseudomallei* strain 115 | Present | Present |
|  | *B. pseudomallei* strain C33 | Present | Present |
|  | *B. pseudomallei* strain EY7 | Present | Present |
|  | *B. pseudomallei* strain 4090-0390 | Present | Present |
|  | *B. pseudomallei* strain P157/04 | Present | Present |
|  | *B. pseudomallei* strain 48 | Present | Present |
|  | *B. pseudomallei* strain 708a | Present | Present |
|  | *B. pseudomallei* strain C63 | Present | Present |
|  | *B. pseudomallei* strain C43 | Present | Present |
|  | *B. pseudomallei* strain C56 | Present | Present |
|  | *B. pseudomallei* strain C60 | Present | Present |
|  | *B. pseudomallei* strain 78/96 | Present | Present |
|  | *B. pseudomallei* strain EY5 | Present | Present |
|  | *B. pseudomallei* strain C40 | Present | Present |
|  | *B. pseudomallei* strain C8 | Present | Present |
|  | *B. pseudomallei* strain C45 | Present | Present |
|  | *B. pseudomallei* strain C37 | Present | Present |
|  | *B. pseudomallei* strain C35 | Present | Present |
|  | *B. pseudomallei* strain C24 | Present | Present |
|  | *B. pseudomallei* strain C7 | Present | Present |
|  | *B. pseudomallei* strain C47 | Present | Present |
|  | *B. pseudomallei* 1106b | Present | Present |
|  | *B. pseudomallei* 1710a | Present | Present |
|  | *B. pseudomallei* Bp22 | Present | Present |
|  | *B. pseudomallei* MSHR435 | Present | Present |
|  | *B. pseudomallei* MSHR1079 | Present | Present |
|  | *B. pseudomallei* MSHR1328 | Present | Present |
|  | *B. pseudomallei* strain MSHR6522 | Present | Present |
|  | *B. pseudomallei* NCTC 13178 | Present | Present |
|  | *B. pseudomallei* NAU20B-16 | Present | Present |
|  | *B. pseudomallei* MSHR511 | Present | Present |
|  | *B. pseudomallei* MSHR146 | Present | Present |
|  | *B. pseudomallei* MSHR520 | Present | Present |
|  | *B. pseudomallei* NAU35A-3 | Present | Present |
|  | *B. pseudomallei* 1026b | Present | Present |
|  | *B. pseudomallei* strain BEK | Present | Present |
|  | *B. pseudomallei* strain 1106a | Present | Present |
|  | *B. pseudomallei* strain MSHR346 | Present | Present |
|  | *B. pseudomallei* 576 | Present | Present |
|  | *B. pseudomallei* strain MSHR1655 | Present | Present |
|  | *B. pseudomallei* strain Mahidol-1106a | Present | Present |
|  | *B. pseudomallei* MSHR5855 | Present | Present |
|  | *B. pseudomallei* strain BGR | Present | Present |
|  | *B. pseudomallei* MSHR5858 | Present | Present |
|  | *B. pseudomallei* HBPUB10303a | Present | Present |
|  | *B. pseudomallei* MSHR5848 | Present | Present |
|  | *B. pseudomallei* HBPUB10134a | Present | Present |
|  | *B. pseudomallei* strain BGK | Present | Present |
|  | *B. pseudomallei* strain BSR | Present | Present |
|  | *B. pseudomallei* B03 | Present | Present |
|  | *B. pseudomallei* MSHR3965 | Present | Present |
|  | *B. pseudomallei* strain TSV202 | Present | Present |
|  | *B. pseudomallei* TSV 48 | Present | Present |
|  | *B. pseudomallei* K42 | Present | Present |
|  | *B. pseudomallei* A79A | Present | Present |
|  | *B. pseudomallei* strain BDP | Present | Present |
|  | *B. pseudomallei* MSHR62 | Present | Present |
|  | *B. pseudomallei* MSHR2243 | Present | Present |
|  | *B. pseudomallei* MSHR1153 | Present | Present |
|  | *B. pseudomallei* 406e | Present | Present |
|  | *B. pseudomallei* MSHR840 | Present | Present |
|  | *B. pseudomallei* MSHR2543 | Present | Present |
|  | *B. pseudomallei* MSHR491 | Present | Present |
|  | *B. pseudomallei* 7894 | Present | Present |
|  | *B. pseudomallei* K96243 (NZ_CP009537) | Present | Present |
|  | *B. pseudomallei* strain MSHR668 | Present | Present |
|  | *B. pseudomallei* PB08298010 | Present | Present |
|  | *B. pseudomallei* strain PHLS 112 | Present | Present |
|  | *B. pseudomallei* Pasteur 52237 | Present | Present |
|  | *B. pseudomallei* strain vgh07 | Present | Present |
|  | *B. pseudomallei* strain Bp1651 | Present | Present |
|  | *B. pseudomallei* strain vgh16R | Present | Present |
|  | *B. pseudomallei* strain vgh16W | Present | Present |
|  | *B. pseudomallei* strain 982 | Present | Present |
|  | *B. pseudomallei* strain MS | Present | Present |
|  | *B. pseudomallei* strain M1 | Present | Present |
|  | *B. pseudomallei* strain Burk178-Type1 | Present | Present |
|  | *B. pseudomallei* strain Burk178-Type2 | Present | Present |
|  | *B. pseudomallei* strain MSHR7929 | Present | Present |
|  | *B. pseudomallei* strain MSHR6755 | Present | Present |
|  | *B. pseudomallei* strain MSHR5864 | Present | Present |
|  | *B. pseudomallei* strain MSHR4083 | Present | Present |
|  | *B. pseudomallei* strain MSHR3763 | Present | Present |
|  | *B. pseudomallei* strain VB976100 | Present | Present |
|  | *B. pseudomallei* strain 2002721100 | Present | Present |
|  | *B. pseudomallei* strain 2002721123 | Present | Present |
|  | *B. pseudomallei* strain 2002721171 | Present | Present |
|  | *B. pseudomallei* strain 2002721184 | Present | Present |
|  | *B. pseudomallei* strain 2002721684 | Present | Present |
|  | *B. pseudomallei* strain 2008724734 | Present | Present |
|  | *B. pseudomallei* strain 2008724758 | Present | Present |
|  | *B. pseudomallei* strain 2008724860 | Present | Present |
|  | *B. pseudomallei* strain 2010007509 | Present | Present |
|  | *B. pseudomallei* strain 2011756189 | Present | Present |
|  | *B. pseudomallei* strain 2011756295 | Present | Present |
|  | *B. pseudomallei* strain 2011756296 | Present | Present |
|  | *B. pseudomallei* strain 2013746776 | Present | Present |
|  | *B. pseudomallei* strain 2013746777 | Present | Present |
|  | *B. pseudomallei* strain 2013746811 | Present | Present |
|  | *B. pseudomallei* strain 2013746877 | Present | Present |
|  | *B. pseudomallei* strain 2013746878 | Present | Present |
|  | *B. pseudomallei* strain 2013833055 | Present | Present |
|  | *B. pseudomallei* strain 2013833057 | Present | Present |
|  | *B. pseudomallei* strain 3000015237 | Present | Present |
|  | *B. pseudomallei* strain 3000015486 | Present | Present |
|  | *B. pseudomallei* strain 3000047530 | Present | Present |
|  | *B. pseudomallei* strain 3000465972 | Present | Present |
|  | *B. pseudomallei* strain 2002734728 | Present | Present |
|  | *B. pseudomallei* strain 14M0960418 | Present | Present |
|  | *B. pseudomallei* strain BPHN1 | Present | Present |
|  | *B. pseudomallei* strain MSHR1435 | Present | Present |
|  | *B. pseudomallei* strain H10 | Present | Present |
|  | *B. pseudomallei* strain PMC2000 | Present | Present |
|  | *B. pseudomallei* strain R15 | Present | Present |
|  | *B. pseudomallei* strain D286 | Present | Present |
|  | *B. pseudomallei* strain FDAARGOS 594 | Present | Present |
|  | *B. pseudomallei* strain FDAARGOS 593 | Present | Present |
|  | *B. pseudomallei* strain FDAARGOS 592 | Present | Present |
|  | *B. pseudomallei* strain BPs110 | Present | Present |
|  | *B. pseudomallei* strain BPs111 | Present | Present |
|  | *B. pseudomallei* strain BPs115 | Present | Present |
|  | *B. pseudomallei* strain BPs116 | Present | Present |
|  | *B. pseudomallei* strain BPs123 | Present | Present |
|  | *B. pseudomallei* strain BPs133 | Present | Present |
|  | *B. pseudomallei* strain BPs114 | Present | Present |
|  | *B. pseudomallei* strain BPs112 | Present | Present |
|  | *B. pseudomallei* strain BPs122 | Present | Present |
|  | *B. pseudomallei* strain Yap6 | Present | Present |
|  | *B. pseudomallei* strain Yap7 | Present | Present |
|  | *B. pseudomallei* strain Yap5 | Present | Present |
|  | *B. pseudomallei* strain Yap4 | Present | Present |
|  | *B. pseudomallei* strain Yap3a | Present | Present |
|  | *B. pseudomallei* strain Yap2a | Present | Present |
|  | *B. pseudomallei* strain Yap1 | Present | Present |
|  | *B. pseudomallei* strain HNBP001 | Present | Present |
|  | *B. pseudomallei* strain InDRE 1Son2018 | Present | Present |
|  | *B. pseudomallei* strain InDRE 2Son2018 | Present | Present |
|  | *B. pseudomallei* strain B03 | Present | Present |
|  | *B. pseudomallei* strain VB29710 | Present | Present |
|  | *B. pseudomallei* strain VBM21822 | Present | Present |
|  | *B. pseudomallei* strain VBM21885 | Present | Present |
|  | *B. pseudomallei* strain VBM23831 | Present | Present |
|  | *B. pseudomallei* strain VBP364 | Present | Present |
|  | *B. pseudomallei* strain VBP399 | Present | Present |
|  | *B. pseudomallei* strain VBP21885 | Present | Present |
|  | *B. pseudomallei* strain VBP23831 | Present | Present |
|  | *B. pseudomallei* strain WC19 | Present | Present |
|  | *B. pseudomallei* strain N2 | Present | Present |
|  | *B. pseudomallei* strain BD5 | Present | Present |
|  | *B. pseudomallei* strain AW44 | Present | Present |
|  | *B. pseudomallei* strain AW17-23 | Present | Present |
|  | *B. pseudomallei* strain AW17-22 | Present | Present |
|  | *B. pseudomallei* strain AW9 | Present | Present |
|  | *B. pseudomallei* strain VBP21822 | Present | Present |
|  | *B. pseudomallei* strain MSHR7744 | Present | Present |
|  | *B. pseudomallei* strain MSHR1713 | Present | Present |
|  | *B. pseudomallei* strain MSHR1046 | Present | Present |
|  | *B. pseudomallei* strain MSHR5087 | Present | Present |
|  | *B. pseudomallei* strain MSHR5089 | Present | Present |
|  | *B. pseudomallei* strain 8 | Present | Present |
|  | *B. pseudomallei* strain 17 | Present | Present |
|  | *B. pseudomallei* strain 56 | Present | Present |
|  | *B. pseudomallei* strain 4033-10 | Present | Present |
|  | *B. pseudomallei* strain 4044-138 | Present | Present |
|  | *B. pseudomallei* strain 4086-21 | Present | Present |
|  | *B. pseudomallei* strain 4094-225 | Present | Present |
|  | *B. pseudomallei* strain D1 9033-5008 | Present | Present |
|  | *B. pseudomallei* strain D7 3230-3018 | Present | Present |
|  | *B. pseudomallei* strain DB19897/02 | Present | Present |
|  | *B. pseudomallei* strain DR08726/01 | Present | Present |
|  | *B. pseudomallei* strain DB61901/00 | Present | Present |
|  | *B. pseudomallei* strain EB 6103/04 | Present | Present |
|  | *B. pseudomallei* strain EY1 | Present | Present |
|  | *B. pseudomallei* strain DR13450/01 | Present | Present |
|  | *B. pseudomallei* strain EY2 | Present | Present |
|  | *B. pseudomallei* strain EY3 | Present | Present |
|  | *B. pseudomallei* strain 107 | Present | Present |
|  | *B. pseudomallei* strain 488 | Present | Present |
|  | *B. pseudomallei* strain 15/96 | Present | Present |
|  | *B. pseudomallei* strain K1127 7244-293 | Present | Present |
|  | *B. pseudomallei* strain 10./96 | Present | Present |
|  | *B. pseudomallei* strain 21/96 | Present | Present |
|  | *B. pseudomallei* strain 561 | Present | Present |
|  | *B. pseudomallei* strain 2/96 | Present | Present |
|  | *B. pseudomallei* strain ATCC 15682 | Present | Present |
|  | *B. pseudomallei* strain P164/04 | Present | Present |
|  | *B. pseudomallei* strain 6./96 | Present | Present |
|  | *B. pseudomallei* strain 457/96 | Present | Present |
|  | *B. pseudomallei* strain 504/96 | Present | Present |
|  | *B. pseudomallei* strain P166/04 | Present | Present |
|  | *B. pseudomallei* strain G. Shepherd | Present | Present |
|  | *B. pseudomallei* strain EY10 | Present | Present |
|  | *B. pseudomallei* strain P163/04 | Present | Present |
|  | *B. pseudomallei* strain P165/04 | Present | Present |
|  | *B. pseudomallei* strain P6 | Present | Present |
|  | *B. pseudomallei* strain P169/04 | Present | Present |
|  | *B. pseudomallei* strain P171/04 | Present | Present |
|  | *B. pseudomallei* strain P170/04 | Present | Present |
|  | *B. pseudomallei* strain 12-40 | Present | Present |
|  | *B. pseudomallei* strain 15-40 | Present | Present |
|  | *B. pseudomallei* strain SW9 | Present | Present |
|  | *B. pseudomallei* strain E25 | Present | Present |
|  | *B. pseudomallei* strain C48 | Present | Present |
|  | *B. pseudomallei* strain C54 | Present | Present |
|  | *B. pseudomallei* strain C51 | Present | Present |
|  | *B. pseudomallei* strain C3 | Present | Present |
|  | *B. pseudomallei* strain C17 | Present | Present |
|  | *B. pseudomallei* strain C31 | Present | Present |
|  | *B. pseudomallei* strain C50 | Present | Present |
|  | *B. pseudomallei* strain C22 | Present | Present |
|  | *B. pseudomallei* strain C68 | Present | Present |
|  | *B. pseudomallei* strain C52 | Present | Present |
|  | *B. pseudomallei* strain C26 | Present | Present |
|  | *B. pseudomallei* strain C42 | Present | Present |
|  | *B. pseudomallei* strain C21 | Present | Present |
|  | *B. pseudomallei* strain C70 | Present | Present |
|  | *B. pseudomallei* strain C58 | Present | Present |
|  | *B. pseudomallei* strain C25 | Present | Present |
|  | *B. pseudomallei* strain C15 | Present | Present |
|  | *B. pseudomallei* strain C1 | Present | Present |
|  | *B. pseudomallei* strain C62 | Present | Present |
|  | *B. pseudomallei* strain C69 | Present | Present |
|  | *B. pseudomallei* strain C66 | Present | Present |
|  | *B. pseudomallei* strain C29 | Present | Present |
|  | *B. pseudomallei* strain C32 | Present | Present |
|  | *B. pseudomallei* strain C36 | Present | Present |
|  | *B. pseudomallei* strain C16 | Present | Present |
|  | *B. pseudomallei* strain C10 | Present | Present |
|  | *B. pseudomallei* strain C53 | Present | Present |
|  | *B. pseudomallei* strain C11 | Present | Present |
|  | *B. pseudomallei* strain C28 | Present | Present |
|  | *B. pseudomallei* strain C30 | Present | Present |
|  | *B. pseudomallei* strain C13 | Present | Present |
|  | *B. pseudomallei* strain C44 | Present | Present |
|  | *B. pseudomallei* strain C2 | Present | Present |
|  | *B. pseudomallei* strain 23/96 | Present | Present |
|  | *B. pseudomallei* strain ATCC 23343 | Present | Present |
|  | *B. pseudomallei* strain HA | Present | Present |
|  | *B. pseudomallei* strain EY6 | Present | Present |
|  | *B. pseudomallei* strain 413 | Present | Present |
|  | *B. pseudomallei* strain 147 | Present | Present |
|  | *B. pseudomallei* strain Bp1774 | Present | Present |
|  | *B. pseudomallei* strain Bp1927 | Present | Present |
|  | *B. pseudomallei* strain Bp1810 | Present | Present |
|  | *B. pseudomallei* strain Bp8880 | Present | Present |
|  | *B. pseudomallei* strain Bp8870 | Present | Present |
|  | *B. pseudomallei* strain Bp8887 | Present | Present |
|  | *B. pseudomallei* strain Bp8877 | Present | Present |
|  | *B. pseudomallei* strain Bp8875 | Present | Present |
|  | *B. pseudomallei* strain Bp8889 | Present | Present |
|  | *B. pseudomallei* strain Bp8891 | Present | Present |
|  | *B. pseudomallei* strain Bp8881 | Present | Present |
|  | *B. pseudomallei* strain Bp8883 | Present | Present |
|  | *B. pseudomallei* strain Bp8866 | Present | Present |
|  | *B. pseudomallei* strain Bp8869 | Present | Present |
|  | *B. pseudomallei* strain Bp8878 | Present | Present |
|  | *B. pseudomallei* strain Bp8879 | Present | Present |
|  | *B. pseudomallei* strain Bp8885 | Present | Present |
|  | *B. pseudomallei* strain Bp8872 | Present | Present |
|  | *B. pseudomallei* strain Bp8884 | Present | Present |
|  | *B. pseudomallei* strain Bp8890 | Present | Present |
|  | *B. pseudomallei* strain Bp8882 | Present | Present |
|  | *B. pseudomallei* strain Bp8886 | Present | Present |
|  | *B. pseudomallei* strain Bp8892 | Present | Present |
|  | *B. pseudomallei* strain Bp8871 | Present | Present |
|  | *B. pseudomallei* strain Bp8894 | Present | Present |
|  | *B. pseudomallei* strain haikou1 | Present | Present |
|  | *B. pseudomallei* strain haikou2 | Present | Present |
|  | *B. pseudomallei* strain haikou3 | Present | Present |
|  | *B. pseudomallei* strain haikou4 | Present | Present |
|  | *B. pseudomallei* strain haikou5 | Present | Present |
|  | *B. pseudomallei* strain haikou6 | Present | Present |
|  | *B. pseudomallei* strain haikou7 | Present | Present |
|  | *B. pseudomallei* strain haikou8 | Present | Present |
|  | *B. pseudomallei* strain 91W S76 | Present | Present |
|  | *B. pseudomallei* strain 64W S75 | Present | Present |
|  | *B. pseudomallei* strain 60W S74 | Present | Present |
|  | *B. pseudomallei* strain 58W S73 | Present | Present |
|  | *B. pseudomallei* strain 53W S72 | Present | Present |
|  | *B. pseudomallei* strain 52W S71 | Present | Present |
|  | *B. pseudomallei* strain 51W S70 | Present | Present |
|  | *B. pseudomallei* strain 50W S69 | Present | Present |
|  | *B. pseudomallei* strain 49W S68 | Present | Present |
|  | *B. pseudomallei* strain 46W S66 | Present | Present |
|  | *B. pseudomallei* strain 47W S67 | Present | Present |
|  | *B. pseudomallei* strain 45S S65 | Present | Present |
|  | *B. pseudomallei* strain 44W S64 | Present | Present |
|  | *B. pseudomallei* strain 43S S63 | Present | Present |
|  | *B. pseudomallei* strain 43W S62 | Present | Present |
|  | *B. pseudomallei* strain 40S S61 | Present | Present |
|  | *B. pseudomallei* strain 294H | Present | Present |
|  | *B. pseudomallei* strain VB30019 | Present | Present |
|  | *B. pseudomallei* strain VB23014 | Present | Present |
|  | *B. pseudomallei* strain VB21320 | Present | Present |
|  | *B. pseudomallei* strain VB20979 | Present | Present |
|  | *B. pseudomallei* strain VB19515 | Present | Present |
|  | *B. pseudomallei* strain VB19512 | Present | Present |
|  | *B. pseudomallei* strain VB7468 | Present | Present |
|  | *B. pseudomallei* strain VB5829 | Present | Present |
|  | *B. pseudomallei* strain VB260 | Present | Present |
|  | *B. pseudomallei* strain VB23080 | Present | Present |
|  | *B. pseudomallei* strain VB20536 | Present | Present |
|  | *B. pseudomallei* strain VB2447 | Present | Present |
|  | *B. pseudomallei* strain VB2691 | Present | Present |
|  | *B. pseudomallei* strain VB3950 | Present | Present |
|  | *B. pseudomallei* strain VB3719 | Present | Present |
|  | *B. pseudomallei* strain VB380 | Present | Present |
|  | *B. pseudomallei* strain VB357 | Present | Present |
|  | *B. pseudomallei* strain VB109 | Present | Present |
|  | *B. pseudomallei* strain VB41637 | Present | Present |
|  | *B. pseudomallei* strain VB41042 | Present | Present |
|  | *B. pseudomallei* strain VB40892 | Present | Present |
|  | *B. pseudomallei* strain VB39505 | Present | Present |
|  | *B. pseudomallei* strain VB39424 | Present | Present |
|  | *B. pseudomallei* strain VB36011 | Present | Present |
|  | *B. pseudomallei* strain VB35420 | Present | Present |
|  | *B. pseudomallei* strain VB35033 | Present | Present |
|  | *B. pseudomallei* strain VB28189 | Present | Present |
|  | *B. pseudomallei* strain VB23841 | Present | Present |
|  | *B. pseudomallei* strain VB23722 | Present | Present |
|  | *B. pseudomallei* strain VB19309 | Present | Present |
|  | *B. pseudomallei* strain VB19166 | Present | Present |
|  | *B. pseudomallei* strain VB18878 | Present | Present |
|  | *B. pseudomallei* strain VB18845 | Present | Present |
|  | *B. pseudomallei* strain VB17718 | Present | Present |
|  | *B. pseudomallei* strain VB17632 | Present | Present |
|  | *B. pseudomallei* strain VB17260 | Present | Present |
|  | *B. pseudomallei* strain VB17203 | Present | Present |
|  | *B. pseudomallei* strain VB15671 | Present | Present |
|  | *B. pseudomallei* strain VB4492 | Present | Present |
|  | *B. pseudomallei* strain VB25647 | Present | Present |
|  | *B. pseudomallei* strain Bp8874 | Present | Present |
|  | *B. pseudomallei* strain AW21 | Present | Present |
|  | *B. pseudomallei* strain BpTX2021NHP | Present | Present |
|  | *B. pseudomallei* strain BpTX2015NHP | Present | Present |
|  | *B. pseudomallei* strain BpTX2014NHP | Present | Present |
|  | *B. pseudomallei* strain qseBC | Present | Present |
|  | *B. pseudomallei* strain Mayo G014009607 | Present | Present |
|  | *B. pseudomallei* strain BPK2 utg000002l polish | Present | Present |
|  | *B. pseudomallei* strain BPK1 | Present | Present |
|  | *B. pseudomallei* strain Maran | Present | Present |
|  | *B. pseudomallei* strain UMC067 | Present | Present |
|  | *B. pseudomallei* strain UMC070 | Present | Present |
|  | *B. pseudomallei* strain CM000113 | Present | Present |
|  | *B. pseudomallei* strain 9501KV | Present | Present |
|  | *B. pseudomallei* strain 2119B | Present | Present |
|  | *B. pseudomallei* strain 21198 | Present | Present |
|  | *B. pseudomallei* strain 21013 | Present | Present |
|  | *B. pseudomallei* strain 28P | Present | Present |
|  | *B. pseudomallei* strain 284 | Present | Present |
|  | *B. pseudomallei* strain 2125 | Present | Present |
|  | *B. pseudomallei* strain VPKA2 | Present | Present |
|  | *B. pseudomallei* strain VP07 | Present | Present |
|  | *B. pseudomallei* strain VP03 | Present | Present |
|  | *B. pseudomallei* strain VP200 | Present | Present |
|  | *B. pseudomallei* strain VP161 | Present | Present |
|  | *B. pseudomallei* strain VP069 | Present | Present |
|  | *B. pseudomallei* strain VP044 | Present | Present |
|  | *B. pseudomallei* strain 9601 | Present | Present |
|  | *B. pseudomallei* strain 9501 | Present | Present |
|  | *B. pseudomallei* strain 9500 | Present | Present |
|  | *B. pseudomallei* strain 8400 | Present | Present |
|  | *B. pseudomallei* strain 7803 | Present | Present |
|  | *B. pseudomallei* strain 7801 | Present | Present |
|  | *B. pseudomallei* strain 7403 | Present | Present |
|  | *B. pseudomallei* strain 7400 | Present | Present |
|  | *B. pseudomallei* strain PT03 | Present | Present |
|  | *B. pseudomallei* strain YB16 | Present | Present |
|  | *B. pseudomallei* strain 128 | Present | Present |
|  | *B. pseudomallei* strain 118-2 | Present | Present |
|  | *B. pseudomallei* strain 111-2 | Present | Present |
|  | *B. pseudomallei* strain EVN00001 | Present | Present |
|  | *B. pseudomallei* strain 22MB038115 | Present | Present |
|  | *B. pseudomallei* strain 22MB037893 | Present | Present |
|  | *B. pseudomallei* strain 22MB259711 | Present | Present |
|  | *B. pseudomallei* strain 22MB696468 | Present | Present |
|  | *B. pseudomallei* strain 22MB035840 | Present | Present |
|  | *B. pseudomallei* strain 22MB088990 | Present | Present |
|  | *B. pseudomallei* strain 22MB088399 | Present | Present |
|  | *B. pseudomallei* strain 22MB033903 | Present | Present |
|  | *B. pseudomallei* strain 22MB033713 | Present | Present |
|  | *B. pseudomallei* strain 22MB688185 | Present | Present |
|  | *B. pseudomallei* strain 22MB031188 | Present | Present |
|  | *B. pseudomallei* strain 22MB028960 | Present | Present |
|  | *B. pseudomallei* strain 22MB253551 | Present | Present |
|  | *B. pseudomallei* strain 22MB028671 | Present | Present |
|  | *B. pseudomallei* strain 22MB028129 | Present | Present |
|  | *B. pseudomallei* strain 22MB028052 | Present | Present |
|  | *B. pseudomallei* strain 22MB028029 | Present | Present |
|  | *B. pseudomallei* strain 22MB252673 | Present | Present |
|  | *B. pseudomallei* strain 22MB073876 | Present | Present |
|  | *B. pseudomallei* strain 22MB014009 | Present | Present |
|  | *B. pseudomallei* strain 22M0561396 | Present | Present |
|  | *B. pseudomallei* strain 22MB003654 | Present | Present |
|  | *B. pseudomallei* strain 21MB038740 | Present | Present |
|  | *B. pseudomallei* strain 21MB264425 | Present | Present |
|  | *B. pseudomallei* strain 21MB026222 | Present | Present |
|  | *B. pseudomallei* strain 20MB027741 | Present | Present |
|  | *B. pseudomallei* strain 20MB036336 | Present | Present |
|  | *B. pseudomallei* strain 20MB029364 | Present | Present |
|  | *B. pseudomallei* strain 19MB030927 | Present | Present |
|  | *B. pseudomallei* strain 19MB054434 | Present | Present |
|  | *B. pseudomallei* strain 18MB023849 | Present | Present |
|  | *B. pseudomallei* strain 18MB037000 | Present | Present |
|  | *B. pseudomallei* strain 18MB086927 | Present | Present |
|  | *B. pseudomallei* strain 17MB032174 | Present | Present |
|  | *B. pseudomallei* strain 16MB038851 | Present | Present |
|  | *B. pseudomallei* strain 15MB028567 | Present | Present |
|  | *B. pseudomallei* strain MSHR5091 | Present | Present |
|  | *B. pseudomallei* strain BP-1 | Present | Present |
|  | *B. pseudomallei* strain BP-2 | Present | Present |
|  | *B. pseudomallei* strain BP-3 | Present | Present |
|  | *B. pseudomallei* strain BP-4 | Present | Present |
|  | *B. pseudomallei* strain BP-5 | Present | Present |
|  | *B. pseudomallei* strain BP-6 | Present | Present |
|  | *B. pseudomallei* strain BP-7 | Present | Present |
|  | *B. pseudomallei* strain BP-8 | Present | Present |
|  | *B. pseudomallei* strain BP-9 | Present | Present |
|  | *B. pseudomallei* strain BP-10 | Present | Present |
|  | *B. pseudomallei* strain BP-11 | Present | Present |
|  | *B. pseudomallei* strain BP-12 | Present | Present |
|  | *B. pseudomallei* strain BP-13 | Present | Present |
|  | *B. pseudomallei* MSHR296 | Present | Present |
|  | *B. pseudomallei* MSHR487 | Present | Present |
|  | *B. pseudomallei* MSHR503 | Present | Present |
|  | *B. pseudomallei* RF43Bp22 | Present | Present |
|  | *B. pseudomallei* DL36 | Present | Present |
|  | *B. pseudomallei* MSHR139 | Present | Present |
|  | *B. pseudomallei* Gu2143a | Present | Present |
|  | *B. pseudomallei* MSHR338 | Present | Present |
|  | *B. pseudomallei* MSHR730 | Present | Present |
|  | *B. pseudomallei* MSHR2543 | Present | Present |
|  | *B. pseudomallei* MSHR491 | Present | Present |
|  | *B. pseudomallei* MSHR511 | Present | Present |
|  | *B. pseudomallei* MSHR146 | Present | Present |
|  | *B. pseudomallei* NAU35A3 | Present | Present |
|  | *B. pseudomallei* 319a | Present | Present |
|  | *B. pseudomallei* DL28 | Present | Present |
|  | *B. pseudomallei* MSHR800 | Present | Present |
|  | *B. pseudomallei* DL2 | Present | Present |
|  | *B. pseudomallei* DL35 | Present | Present |
|  | *B. pseudomallei* MSHR87 | Present | Present |
|  | *B. pseudomallei* DL17 | Present | Present |
|  | *B. pseudomallei* DL34 | Present | Present |
|  | *B. pseudomallei* INT4-Bp18 | Present | Present |
|  | *B. pseudomallei* STW 214 | Present | Present |
|  | *B. pseudomallei* USAMRU Malaysia 17 | Present | Present |
|  | *B. pseudomallei* Smith 002025 | Present | Present |
|  | *B. pseudomallei* NAU2B9 | Present | Present |
|  | *B. pseudomallei* NAU14B1 | Present | Present |
|  | *B. pseudomallei* MSHR840 | Present | Present |
|  | *B. pseudomallei* MSHR952 | Present | Present |
|  | *B. pseudomallei* MSHR2053 | Present | Present |
|  | *B. pseudomallei* INT2-Bp24 | Present | Present |
|  | *B. pseudomallei* INT2-Bp91 | Present | Present |
|  | *B. pseudomallei* INT2-Bp184 | Present | Present |
|  | *B. pseudomallei* MSHR1079 | Present | Present |
|  | *B. pseudomallei* MSHR5855 | Present | Present |
|  | *B. pseudomallei* strain PtBps01 | Present | Present |
|  | *B. pseudomallei* MSHR5608 | Present | Present |
|  | *B. pseudomallei* MSHR7504 | Present | Present |
|  | *B. pseudomallei* MSHR7527 | Present | Present |
|  | *B. pseudomallei* MSHR5596 | Present | Present |
|  | *B. pseudomallei* MSHR7334 | Present | Present |
|  | *B. pseudomallei* MSHR7498 | Present | Present |
|  | *B. pseudomallei* MSHR5609 | Present | Present |
|  | *B. pseudomallei* MSHR5613 | Present | Present |
|  | *B. pseudomallei* MSHR5569 | Present | Present |
|  | *B. pseudomallei* MSHR7343 | Present | Present |
|  | *B. pseudomallei* ABCPW 107 | Present | Present |
|  | *B. pseudomallei* MSHR5492 | Present | Present |
|  | *B. pseudomallei* MSHR4378 | Present | Present |
|  | *B. pseudomallei* TSV44 Y024 | Present | Present |
|  | *B. pseudomallei* TSV5 Y027 | Present | Present |
|  | *B. pseudomallei* MSHR4868 | Present | Present |
|  | *B. pseudomallei* A79C X997 | Present | Present |
|  | *B. pseudomallei* A79D Y023 | Present | Present |
|  | *B. pseudomallei* TSV32 Y025 | Present | Present |
|  | *B. pseudomallei* TSV28 Y026 | Present | Present |
|  | *B. pseudomallei* MSHR7500 | Present | Present |
|  | *B. pseudomallei* strain QCMRI BP07 | Present | Present |
|  | *B. pseudomallei* strain QCMRI BP13 | Present | Present |
|  | *B. pseudomallei* strain QCMRI BP18 | Present | Present |
|  | *B. pseudomallei* strain QCMRI BP28 | Present | Present |
|  | *B. pseudomallei* strain QCMRI BP32 | Present | Present |
|  | *B. pseudomallei* strain MSHR5107 | Present | Present |
|  | *B. pseudomallei* strain BEB | Present | Present |
|  | *B. pseudomallei* strain BDD | Present | Present |
|  | *B. pseudomallei* strain BDZ | Present | Present |
|  | *B. pseudomallei* strain BED | Present | Present |
|  | *B. pseudomallei* strain BEF | Present | Present |
|  | *B. pseudomallei* strain BDI | Present | Present |
|  | *B. pseudomallei* strain BDM | Present | Present |
|  | *B. pseudomallei* strain BDE | Present | Present |
|  | *B. pseudomallei* strain BDT | Present | Present |
|  | *B. pseudomallei* strain BFD | Present | Present |
|  | *B. pseudomallei* strain BGH | Present | Present |
|  | *B. pseudomallei* strain BGS | Present | Present |
|  | *B. pseudomallei* strain BGQ | Present | Present |
|  | *B. pseudomallei* strain BEC | Present | Present |
|  | *B. pseudomallei* strain BGJ | Present | Present |
|  | *B. pseudomallei* strain BEG | Present | Present |
|  | *B. pseudomallei* strain BEH | Present | Present |
|  | *B. pseudomallei* strain BES | Present | Present |
|  | *B. pseudomallei* strain PFGE B T6 | Present | Present |
|  | *B. pseudomallei* MSHR4304 | Present | Present |
|  | *B. pseudomallei* MSHR543 | Present | Present |
|  | *B. pseudomallei* MSHR983 | Present | Present |
|  | *B. pseudomallei* MSHR465J | Present | Present |
|  | *B. pseudomallei* MSHR4377 | Present | Present |
|  | *B. pseudomallei* MSHR4300 | Present | Present |
|  | *B. pseudomallei* MSHR4372 | Present | Present |
|  | *B. pseudomallei* TSV 43 | Present | Present |
|  | *B. pseudomallei* MSHR4503 | Present | Present |
|  | *B. pseudomallei* MSHR4462 | Present | Present |
|  | *B. pseudomallei* MSHR4032 | Present | Present |
|  | *B. pseudomallei* MSHR4003 | Present | Present |
|  | *B. pseudomallei* MSHR3951 | Present | Present |
|  | *B. pseudomallei* MSHR4308 | Present | Present |
|  | *B. pseudomallei* ABCPW 91 | Present | Present |
|  | *B. pseudomallei* TSV 31 | Present | Present |
|  | *B. pseudomallei* MSHR4299 | Present | Present |
|  | *B. pseudomallei* MSHR3964 | Present | Present |
|  | *B. pseudomallei* ABCPW 30 | Present | Present |
|  | *B. pseudomallei* BDU 2 | Present | Present |
|  | *B. pseudomallei* MSHR3960 | Present | Present |
|  | *B. pseudomallei* MSHR4012 | Present | Present |
|  | *B. pseudomallei* MSHR4375 | Present | Present |
|  | *B. pseudomallei* TSV 25 | Present | Present |
|  | *B. pseudomallei* MSHR4303 | Present | Present |
|  | *B. pseudomallei* MSHR4000 | Present | Present |
|  | *B. pseudomallei* MSHR684 | Present | Present |
|  | *B. pseudomallei* MSHR3458 | Present | Present |
|  | *B. pseudomallei* MSHR303 | Present | Present |
|  | *B. pseudomallei* MSHR2990 | Present | Present |
|  | *B. pseudomallei* MSHR1357 | Present | Present |
|  | *B. pseudomallei* MSHR2451 | Present | Present |
|  | *B. pseudomallei* MSHR3016 | Present | Present |
|  | *B. pseudomallei* MSHR733 | Present | Present |
|  | *B. pseudomallei* MSHR1000 | Present | Present |
|  | *B. pseudomallei* MSHR640 | Present | Present |
|  | *B. pseudomallei* MSHR456 | Present | Present |
|  | *B. pseudomallei* MSHR449 | Present | Present |
|  | *B. pseudomallei* MSHR332 | Present | Present |
|  | *B. pseudomallei* strain MSHR4018 | Present | Present |
|  | *B. pseudomallei* strain MSHR4009 | Present | Present |
|  | *B. pseudomallei* ABCPW 1 | Present | Present |
|  | *B. pseudomallei* strain MSHR44 | Present | Present |
|  | *B. pseudomallei* MSHR3709 | Present | Present |
|  | *B. pseudomallei* MSHR3335 | Present | Present |
|  | *B. pseudomallei* MSHR2138 | Present | Present |
|  | *B. pseudomallei* MSHR1029 | Present | Present |
|  | *B. pseudomallei* strain MSHR0169 | Present | Present |
|  | *B. pseudomallei* strain ST1381 | Present | Present |
|  | *B. pseudomallei* strain C1 | Present | Present |
|  | *B. pseudomallei* isolate UKMH10 | Present | Present |
|  | *B. pseudomallei* isolate UKMPMC2000 | Present | Present |
|  | *B. pseudomallei* isolate UKMD286 | Present | Present |
|  | *B. pseudomallei* isolate UKMR15 | Present | Present |
|  | *B. pseudomallei* strain QCMRI BP11 | Present | Present |
|  | *B. pseudomallei* strain MSHR0913 | Present | Present |
|  | *B. pseudomallei* strain PHLS6 BIM | Present | Present |
|  | *B. pseudomallei* strain 2002721785 | Present | Present |
|  | *B. pseudomallei* strain 2002721738 | Present | Present |
|  | *B. pseudomallei* strain 2002721784 | Present | Present |
|  | *B. pseudomallei* strain 2002721741 | Present | Present |
|  | *B. pseudomallei* strain 2002721740 | Present | Present |
|  | *B. pseudomallei* strain 2002721183 | Present | Present |
|  | *B. pseudomallei* strain 2002721712 | Present | Present |
|  | *B. pseudomallei* strain 2002721787 | Present | Present |
|  | *B. pseudomallei* strain 2002721786 | Present | Present |
|  | *B. pseudomallei* strain 2002721789 | Present | Present |
|  | *B. pseudomallei* strain 2002721772 | Present | Present |
|  | *B. pseudomallei* strain 2002721788 | Present | Present |
|  | *B. pseudomallei* strain 3904b | Present | Present |
|  | *B. pseudomallei* strain 4027a | Present | Present |
|  | *B. pseudomallei* strain 4032a | Present | Present |
|  | *B. pseudomallei* strain 4069a | Present | Present |
|  | *B. pseudomallei* strain 3913a | Present | Present |
|  | *B. pseudomallei* strain 4108a | Present | Present |
|  | *B. pseudomallei* strain 4112a | Present | Present |
|  | *B. pseudomallei* strain 4114a | Present | Present |
|  | *B. pseudomallei* strain 4147a | Present | Present |
|  | *B. pseudomallei* strain 4151a | Present | Present |
|  | *B. pseudomallei* strain 3943a | Present | Present |
|  | *B. pseudomallei* strain 4263b | Present | Present |
|  | *B. pseudomallei* strain 4166a | Present | Present |
|  | *B. pseudomallei* strain 4169d | Present | Present |
|  | *B. pseudomallei* strain 4175a | Present | Present |
|  | *B. pseudomallei* strain 4179a | Present | Present |
|  | *B. pseudomallei* strain 4187a | Present | Present |
|  | *B. pseudomallei* strain 4191a | Present | Present |
|  | *B. pseudomallei* strain 4210a | Present | Present |
|  | *B. pseudomallei* strain 3944b | Present | Present |
|  | *B. pseudomallei* strain 4228b | Present | Present |
|  | *B. pseudomallei* strain 3997a | Present | Present |
|  | *B. pseudomallei* strain RF4-BP80 | Present | Present |
|  | *B. pseudomallei* strain RF6-BP2 | Present | Present |
|  | *B. pseudomallei* strain RF6-BP15 | Present | Present |
|  | *B. pseudomallei* strain RF6-BP26 | Present | Present |
|  | *B. pseudomallei* strain RF8-BP2 | Present | Present |
|  | *B. pseudomallei* strain RF8-BP5 | Present | Present |
|  | *B. pseudomallei* strain RF43-BP22 | Present | Present |
|  | *B. pseudomallei* strain RF44-BP52 | Present | Present |
|  | *B. pseudomallei* strain RF49-BP2 | Present | Present |
|  | *B. pseudomallei* strain RF49-BP5 | Present | Present |
|  | *B. pseudomallei* strain RF49-BP7 | Present | Present |
|  | *B. pseudomallei* strain NRF57-BP22 | Present | Present |
|  | *B. pseudomallei* strain NRF57-BP62 | Present | Present |
|  | *B. pseudomallei* strain NRF57-BP64 | Present | Present |
|  | *B. pseudomallei* strain NRF57-BP67 | Present | Present |
|  | *B. pseudomallei* strain NRF60-BP3 | Present | Present |
|  | *B. pseudomallei* strain NRF60-BP4 | Present | Present |
|  | *B. pseudomallei* strain RF61-BP2 | Present | Present |
|  | *B. pseudomallei* strain RF62-BP41 | Present | Present |
|  | *B. pseudomallei* strain RF63-BP1 | Present | Present |
|  | *B. pseudomallei* strain RF63-BP2 | Present | Present |
|  | *B. pseudomallei* strain RF67-BP1 | Present | Present |
|  | *B. pseudomallei* strain RF68-BP5 | Present | Present |
|  | *B. pseudomallei* strain NRF77-BP3 | Present | Present |
|  | *B. pseudomallei* strain SBCT-RF80-BP1 | Present | **Absent** |
|  | *B. pseudomallei* strain RF85-BP37 | Present | Present |
|  | *B. pseudomallei* strain RF87-BP2 | Present | Present |
|  | *B. pseudomallei* strain INT2-BP12 | Present | Present |
|  | *B. pseudomallei* strain INT2-BP24 | Present | Present |
|  | *B. pseudomallei* strain INT2-BP38 | Present | Present |
|  | *B. pseudomallei* strain INT2-BP61 | Present | Present |
|  | *B. pseudomallei* strain INT2-BP89 | Present | Present |
|  | *B. pseudomallei* strain INT2-BP100 | Present | Present |
|  | *B. pseudomallei* strain INT2-BP102 | Present | Present |
|  | *B. pseudomallei* strain INT2-BP105 | Present | Present |
|  | *B. pseudomallei* strain INT2-BP127 | Present | Present |
|  | *B. pseudomallei* strain INT2-BP133 | Present | Present |
|  | *B. pseudomallei* strain INT2-BP175 | Present | Present |
|  | *B. pseudomallei* strain INT2-BP184 | Present | Present |
|  | *B. pseudomallei* strain INT2-BP214 | Present | Present |
|  | *B. pseudomallei* strain INT2-BP217 | Present | Present |
|  | *B. pseudomallei* strain INT2-BP235 | Present | Present |
|  | *B. pseudomallei* strain INT2-BP241 | Present | Present |
|  | *B. pseudomallei* strain INT2-BP264 | Present | Present |
|  | *B. pseudomallei* strain INT4-BP18 | Present | Present |
|  | *B. pseudomallei* strain 319a | Present | Present |
|  | *B. pseudomallei* strain RNS3-Bp1 | Present | Present |
|  | *B. pseudomallei* strain RNS7-Bp6 | Present | Present |
|  | *B. pseudomallei* strain RNS8-BP1 | Present | Present |
|  | *B. pseudomallei* strain Ubon-P19-Bp11 | Present | Present |
|  | *B. pseudomallei* strain Ubon-P19-Bp25 | Present | Present |
|  | *B. pseudomallei* strain Ubon-P19-Bp34 | Present | Present |
|  | *B. pseudomallei* strain Ubon-P19-Bp45 | Present | Present |
|  | *B. pseudomallei* strain Ubon-P19-Bp57 | Present | Present |
|  | *B. pseudomallei* strain Ubon-P23-Bp05 | Present | Present |
|  | *B. pseudomallei* strain Ubon-P23-Bp20 | Present | Present |
|  | *B. pseudomallei* strain Ubon-P23-Bp21 | Present | Present |
|  | *B. pseudomallei* strain Ubon-P23-Bp38 | Present | Present |
|  | *B. pseudomallei* strain Ubon-P44-Bp10 | Present | Present |
|  | *B. pseudomallei* strain Ubon-P44-Bp11 | Present | Present |
|  | *B. pseudomallei* strain Ubon-P44-Bp26 | Present | Present |
|  | *B. pseudomallei* strain Ubon-P44-Bp34 | Present | Present |
|  | *B. pseudomallei* strain Ubon-P45-Bp25 | Present | Present |
|  | *B. pseudomallei* strain Ubon-P45-Bp30 | Present | Present |
|  | *B. pseudomallei* strain MSHR0730 | Present | Present |
|  | *B. pseudomallei* strain MSHR0487 | Present | Present |
|  | *B. pseudomallei* strain MSHR0644 | Present | Present |
|  | *B. pseudomallei* strain MSHR0465A | Present | Present |
|  | *B. pseudomallei* strain MSHR0776 | Present | Present |
|  | *B. pseudomallei* strain MSHR0503 | Present | Present |
|  | *B. pseudomallei* strain NAU2B-1 | Present | Present |
|  | *B. pseudomallei* strain NAU2B-5 | Present | Present |
|  | *B. pseudomallei* strain NAU2B-6 | Present | Present |
|  | *B. pseudomallei* strain NAU2B-8 | Present | Present |
|  | *B. pseudomallei* strain NAU2B-9 | Present | Present |
|  | *B. pseudomallei* strain NAU2B-11 | Present | Present |
|  | *B. pseudomallei* strain NAU2B-12 | Present | Present |
|  | *B. pseudomallei* strain NAU13B-1 | Present | Present |
|  | *B. pseudomallei* strain NAU14A-1 | Present | Present |
|  | *B. pseudomallei* strain NAU14A-2 | Present | Present |
|  | *B. pseudomallei* strain NAU14A-4 | Present | Present |
|  | *B. pseudomallei* strain NAU14A-5 | Present | Present |
|  | *B. pseudomallei* strain NAU14A-8 | Present | Present |
|  | *B. pseudomallei* strain NAU14A-10 | Present | Present |
|  | *B. pseudomallei* strain NAU14B-1 | Present | Present |
|  | *B. pseudomallei* strain NAU14B-2 | Present | Present |
|  | *B. pseudomallei* strain NAU14B-3 | Present | Present |
|  | *B. pseudomallei* strain NAU14B-5 | Present | Present |
|  | *B. pseudomallei* strain NAU14B-6 | Present | Present |
|  | *B. pseudomallei* strain NAU14B-8 | Present | Present |
|  | *B. pseudomallei* strain NAU14B-9 | Present | **Absent** |
|  | *B. pseudomallei* strain NAU14B-10 | Present | Present |
|  | *B. pseudomallei* strain NAU20B-2 | Present | Present |
|  | *B. pseudomallei* strain NAU20B-5 | Present | Present |
|  | *B. pseudomallei* strain NAU20B-8 | Present | Present |
|  | *B. pseudomallei* strain NAU21B-3 | Present | Present |
|  | *B. pseudomallei* strain NAU21B-4 | Present | Present |
|  | *B. pseudomallei* strain NAU21B-7 | Present | Present |
|  | *B. pseudomallei* strain NAU21B-11 | Present | Present |
|  | *B. pseudomallei* strain NAU22A-3 | Present | Present |
|  | *B. pseudomallei* strain NAU22A-5 | Present | Present |
|  | *B. pseudomallei* strain NAU22B-1 | Present | Present |
|  | *B. pseudomallei* strain NAU24B-3 | Present | Present |
|  | *B. pseudomallei* strain NAU33A-6 | Present | Present |
|  | *B. pseudomallei* strain NAU35A-2 | Present | Present |
|  | *B. pseudomallei* strain NAU35A-4 | Present | Present |
|  | *B. pseudomallei* strain NAU35B-2 | Present | Present |
|  | *B. pseudomallei* strain NAU35B-3 | Present | Present |
|  | *B. pseudomallei* strain NAU35B-4 | Present | Present |
|  | *B. pseudomallei* strain NAU44A-2 | Present | Present |
|  | *B. pseudomallei* strain NAU44A-6 | Present | Present |
|  | *B. pseudomallei* strain NAU44A-9 | Present | Present |
|  | *B. pseudomallei* strain MSHR0099 | Present | Present |
|  | *B. pseudomallei* strain MSHR0073 | Present | Present |
|  | *B. pseudomallei* strain MSHR0800 | Present | Present |
|  | *B. pseudomallei* strain MSHR0952 | Present | Present |
|  | *B. pseudomallei* strain MSHR2053 | Present | Present |
|  | *B. pseudomallei* strain MSHR0663 | Present | Present |
|  | *B. pseudomallei* strain MSHR1048 | Present | Present |
|  | *B. pseudomallei* strain MSHR1218 | Present | Present |
|  | *B. pseudomallei* strain MSHR1286 | Present | Present |
|  | *B. pseudomallei* strain MSHR1290 | Present | Present |
|  | *B. pseudomallei* strain MSHR1418 | Present | Present |
|  | *B. pseudomallei* strain MSHR1821 | Present | Present |
|  | *B. pseudomallei* strain MSHR3042 | Present | Present |
|  | *B. pseudomallei* strain MSHR1300 | Present | Present |
|  | *B. pseudomallei* strain MSHR0087 | Present | Present |
|  | *B. pseudomallei* strain MSHR1088 | Present | Present |
|  | *B. pseudomallei* strain MSHR1879 | Present | **Absent** |
|  | *B. pseudomallei* strain MSHR1888 | Present | Present |
|  | *B. pseudomallei* strain GU-2143A | Present | Present |
|  | *B. pseudomallei* strain GU-1909A | Present | Present |
|  | *B. pseudomallei* strain MSHR0376 | Present | Present |
|  | *B. pseudomallei* strain MSHR0391 | Present | Present |
|  | *B. pseudomallei* strain MSHR0443 | Present | Present |
|  | *B. pseudomallei* strain MSHR1660 | Present | Present |
|  | *B. pseudomallei* strain MSHR1670 | Present | Present |
|  | *B. pseudomallei* strain MSHR1672 | Present | Present |
|  | *B. pseudomallei* strain MSHR1676 | Present | Present |
|  | *B. pseudomallei* strain MSHR1677 | Present | Present |
|  | *B. pseudomallei* strain MSHR1678 | Present | Present |
|  | *B. pseudomallei* strain MSHR1679 | Present | Present |
|  | *B. pseudomallei* strain MSHR1682 | Present | Present |
|  | *B. pseudomallei* strain MSHR1683 | Present | Present |
|  | *B. pseudomallei* strain MSHR1684 | Present | Present |
|  | *B. pseudomallei* strain MSHR1685 | Present | Present |
|  | *B. pseudomallei* strain MSHR1688 | Present | Present |
|  | *B. pseudomallei* strain MSHR2845 | Present | Present |
|  | *B. pseudomallei* strain MSHR2849 | Present | Present |
|  | *B. pseudomallei* strain MSHR3030 | Present | Present |
|  | *B. pseudomallei* strain MSHR3106 | Present | Present |
|  | *B. pseudomallei* strain MSHR3107 | Present | Present |
|  | *B. pseudomallei* strain MSHR1289 | Present | Present |
|  | *B. pseudomallei* strain MSHR0200 | Present | Present |
|  | *B. pseudomallei* strain MSHR0232 | Present | Present |
|  | *B. pseudomallei* strain MSHR0295 | Present | Present |
|  | *B. pseudomallei* strain MSHR0347 | Present | Present |
|  | *B. pseudomallei* strain MSHR0474 | Present | Present |
|  | *B. pseudomallei* strain MSHR1079 | Present | Present |
|  | *B. pseudomallei* strain PHLS-9 | Present | Present |
|  | *B. pseudomallei* strain MSHR0120 | Present | Present |
|  | *B. pseudomallei* strain MSHR0296 | Present | Present |
|  | *B. pseudomallei* strain MSHR0445A | Present | Present |
|  | *B. pseudomallei* strain MSHR0911 | Present | Present |
|  | *B. pseudomallei* strain MSHR2825 | Present | Present |
|  | *B. pseudomallei* strain MSHR3499 | Present | Present |
|  | *B. pseudomallei* strain MSHR3662 | Present | Present |
|  | *B. pseudomallei* strain MSHR3841 | Present | Present |
|  | *B. pseudomallei* strain MSHR3876 | Present | Present |
|  | *B. pseudomallei* strain MSHR3974 | Present | Present |
|  | *B. pseudomallei* strain MSHR3998 | Present | Present |
|  | *B. pseudomallei* strain MSHR4250 | Present | Present |
|  | *B. pseudomallei* strain MSHR4301 | Present | Present |
|  | *B. pseudomallei* strain MSHR4483 | Present | Present |
|  | *B. pseudomallei* strain MSHR4504 | Present | Present |
|  | *B. pseudomallei* strain MSHR4637 | Present | Present |
|  | *B. pseudomallei* strain MSHR4638 | Present | Present |
|  | *B. pseudomallei* strain MSHR4735 | Present | Present |
|  | *B. pseudomallei* strain MSHR4736 | Present | Present |
|  | *B. pseudomallei* strain MSHR4737 | Present | Present |
|  | *B. pseudomallei* strain MSHR4738 | Present | Present |
|  | *B. pseudomallei* strain MSHR4749 | Present | Present |
|  | *B. pseudomallei* strain MSHR4750 | Present | Present |
|  | *B. pseudomallei* strain MSHR1141 | Present | Present |
|  | *B. pseudomallei* strain DW10 | Present | Present |
|  | *B. pseudomallei* strain DW7 | Present | Present |
|  | *B. pseudomallei* strain NAU33A-4 | Present | Present |
|  | *B. pseudomallei* strain MSHR5086 | Present | Present |
|  | *B. pseudomallei* strain MSHR5093 | Present | Present |
|  | *B. pseudomallei* strain MSHR5100 | Present | Present |
|  | *B. pseudomallei* strain MSHR5104 | Present | Present |
|  | *B. pseudomallei* strain MSHR5105 | Present | Present |
|  | *B. pseudomallei* strain 5598b | Present | Present |
|  | *B. pseudomallei* strain 5598ci | Present | Present |
|  | *B. pseudomallei* strain 316a | Present | Present |
|  | *B. pseudomallei* strain 316c | Present | Present |
|  | *B. pseudomallei* strain 402a | Present | Present |
|  | *B. pseudomallei* strain 577ci | Present | Present |
|  | *B. pseudomallei* strain 577cii | Present | Present |
|  | *B. pseudomallei* strain 577d | Present | Present |
|  | *B. pseudomallei* strain 858ai | Present | Present |
|  | *B. pseudomallei* strain 858d | Present | Present |
|  | *B. pseudomallei* strain 942a | Present | Present |
|  | *B. pseudomallei* strain 975d | Present | Present |
|  | *B. pseudomallei* strain 979bi | Present | Present |
|  | *B. pseudomallei* strain 979bii | Present | Present |
|  | *B. pseudomallei* strain 984a | Present | Present |
|  | *B. pseudomallei* strain 2374a | Present | Present |
|  | *B. pseudomallei* strain 2374b | Present | Present |
|  | *B. pseudomallei* strain 2381a | Present | Present |
|  | *B. pseudomallei* strain 2381c | Present | Present |
|  | *B. pseudomallei* strain 3013c | Present | Present |
|  | *B. pseudomallei* strain 3964b | Present | Present |
|  | *B. pseudomallei* strain 3964c | Present | Present |
|  | *B. pseudomallei* strain 3964d | Present | Present |
|  | *B. pseudomallei* strain 4095a | Present | Present |
|  | *B. pseudomallei* strain 4226b | Present | Present |
|  | *B. pseudomallei* strain 4226c | Present | Present |
|  | *B. pseudomallei* strain NAU21B-13 | Present | Present |
|  | *B. pseudomallei* strain NAU33A-5 | Present | Present |
|  | *B. pseudomallei* strain NAU35B-7 | Present | Present |
|  | *B. pseudomallei* strain 110 | Present | Present |
|  | *B. pseudomallei* strain 2014002816 | Present | Present |
|  | *B. pseudomallei* strain 2008724644 | Present | Present |
|  | *B. pseudomallei* strain H0901 | Present | Present |
|  | *B. pseudomallei* strain TOML | Present | Present |
|  | *B. pseudomallei* strain TOMS | Present | Present |
|  | *B. pseudomallei* strain VL | Present | Present |
|  | *B. pseudomallei* strain VS | Present | Present |
|  | *B. pseudomallei* strain UM137 | Present | Present |
|  | *B. pseudomallei* strain UM136 | Present | Present |
|  | *B. pseudomallei* strain UM129 | Present | Present |
|  | *B. pseudomallei* strain UM117 | Present | Present |
|  | *B. pseudomallei* strain UMC108 | Present | Present |
|  | *B. pseudomallei* strain UMC107 | Present | Present |
|  | *B. pseudomallei* strain UMC106 | Present | Present |
|  | *B. pseudomallei* strain V1512 | Present | Present |
|  | *B. pseudomallei* strain V1504 | Present | Present |
|  | *B. pseudomallei* strain BP-23 | Present | Present |
|  | *B. pseudomallei* strain BP-3504 | Present | Present |
|  | *B. pseudomallei* strain BP-6260 | Present | Present |
|  | *B. pseudomallei* strain BP-6887 | Present | Present |
|  | *B. pseudomallei* strain ZUSM | **Absent** | Present |
|  | *B. pseudomallei* strain 4811 | Present | Present |
|  | *B. pseudomallei* strain 4802 | Present | Present |
|  | *B. pseudomallei* strain 4811R | Present | Present |
|  | *B. pseudomallei* strain 4802R | Present | Present |
|  | *B. pseudomallei* strain 1701 | Present | Present |
|  | *B. pseudomallei* strain Bp9110 | Present | Present |
|  | *B. pseudomallei* strain Bp9039 | Present | Present |
|  | *B. pseudomallei* strain K96243-Dstl-2 | Present | Present |
|  | *B. pseudomallei* strain K96243-Dstl-1 | Present | Present |
|  | *B. pseudomallei* strain K96243-LSTHM | Present | Present |
|  | *B. pseudomallei* strain K96243-Exeter | Present | Present |
|  | *B. pseudomallei* strain 3001162207 | Present | Present |
|  | *B. pseudomallei* strain 3001161896 | Present | Present |
|  | *B. pseudomallei* strain 3001161892 | Present | Present |
|  | *B. pseudomallei* strain 3001161891 | Present | Present |
|  | *B. pseudomallei* strain FDAARGOS 591 | Present | Present |
|  | *B. pseudomallei* strain Bp9112 | Present | Present |
|  | *B. pseudomallei* strain Bp9050 | Present | Present |
|  | *B. pseudomallei* strain Bp9107 | Present | Present |
|  | *B. pseudomallei* strain JW270 1-JW-270-GDNA NODE | Present | Present |
|  | *B. pseudomallei* strain MAA2018 NODE | Present | Present |
|  | *B. pseudomallei* strain C NODE | Present | Present |
|  | *B. pseudomallei* strain B2 NODE | Present | Present |
|  | *B. pseudomallei* strain B1 NODE | Present | Present |
|  | *B. pseudomallei* strain A2 NODE | Present | Present |
|  | *B. pseudomallei* strain A1 NODE | Present | Present |
|  | *B. pseudomallei* strain Yap3d 2008725079 | Present | Present |
|  | *B. pseudomallei* strain Yap3c 2008725078 | Present | Present |
|  | *B. pseudomallei* strain Yap2e 2008725077 | Present | Present |
|  | *B. pseudomallei* strain Yap3b 2008725076 | Present | Present |
|  | *B. pseudomallei* strain Yap2d 2008725074 | Present | Present |
|  | *B. pseudomallei* strain Yap2c 2008725073 | Present | Present |
|  | *B. pseudomallei* strain Yap2b 2008725072 | Present | Present |
|  | *B. pseudomallei* strain S-547 | Present | Present |
|  | *B. pseudomallei* strain NCTC6700 | Present | Present |
|  | *B. pseudomallei* strain HBPUB10002A | Present | Present |
|  | *B. pseudomallei* strain HBPUB10003A | Present | Present |
|  | *B. pseudomallei* strain HBPUB10004A | Present | Present |
|  | *B. pseudomallei* strain HBPUB10005C | Present | Present |
|  | *B. pseudomallei* strain HBPUB10006A | Present | Present |
|  | *B. pseudomallei* strain HBPUB10008A | Present | Present |
|  | *B. pseudomallei* strain HBPUB10009B | Present | Present |
|  | *B. pseudomallei* strain HBPUB10007A | Present | Present |
|  | *B. pseudomallei* strain HBPUB10010B | Present | Present |
|  | *B. pseudomallei* strain HBPUB10011A | Present | Present |
|  | *B. pseudomallei* strain HBPUB10016A | Present | Present |
|  | *B. pseudomallei* strain HBPUB10013A | Present | Present |
|  | *B. pseudomallei* strain HBPUB10014A | Present | Present |
|  | *B. pseudomallei* strain HBPUB10017A | Present | Present |
|  | *B. pseudomallei* strain HBPUB10018A | Present | Present |
|  | *B. pseudomallei* strain HBPUB10020A | Present | Present |
|  | *B. pseudomallei* strain HBPUB10019A | Present | Present |
|  | *B. pseudomallei* strain HBPUB10021A | Present | Present |
|  | *B. pseudomallei* strain HBPUB10022A | Present | Present |
|  | *B. pseudomallei* strain HBPUB10023A | Present | Present |
|  | *B. pseudomallei* strain HBPUB10026A | Present | Present |
|  | *B. pseudomallei* strain HBPUB10027B | Present | Present |
|  | *B. pseudomallei* strain HBPUB10031C | Present | Present |
|  | *B. pseudomallei* strain HBPUB10024A | Present | Present |
|  | *B. pseudomallei* strain HBPUB10030B | Present | Present |
|  | *B. pseudomallei* strain HBPUB10028A | Present | Present |
|  | *B. pseudomallei* strain HBPUB10032B | Present | Present |
|  | *B. pseudomallei* strain HBPUB10029A | Present | Present |
|  | *B. pseudomallei* strain HBPUB10033A | Present | Present |
|  | *B. pseudomallei* strain HBPUB10034A | Present | Present |
|  | *B. pseudomallei* strain HBPUB10037A | Present | Present |
|  | *B. pseudomallei* strain HBPUB10050F | Present | Present |
|  | *B. pseudomallei* strain HBPUB10055C | Present | Present |
|  | *B. pseudomallei* strain HBPUB10039A | Present | Present |
|  | *B. pseudomallei* strain HBPUB10040A | Present | Present |
|  | *B. pseudomallei* strain HBPUB10035A | Present | Present |
|  | *B. pseudomallei* strain HBPUB10047A | Present | Present |
|  | *B. pseudomallei* strain HBPUB10052A | Present | Present |
|  | *B. pseudomallei* strain HBPUB10051B | Present | Present |
|  | *B. pseudomallei* strain HBPUB10048A | Present | Present |
|  | *B. pseudomallei* strain HBPUB10058A | Present | Present |
|  | *B. pseudomallei* strain HBPUB10056A | Present | Present |
|  | *B. pseudomallei* strain HBPUB10065C | Present | Present |
|  | *B. pseudomallei* strain HBPUB10071C | Present | Present |
|  | *B. pseudomallei* strain HBPUB10059A | Present | Present |
|  | *B. pseudomallei* strain HBPUB10072A | Present | Present |
|  | *B. pseudomallei* strain HBPUB10064A | Present | Present |
|  | *B. pseudomallei* strain HBPUB10061A | Present | Present |
|  | *B. pseudomallei* strain HBPUB10067B | Present | Present |
|  | *B. pseudomallei* strain HBPUB10070B | Present | Present |
|  | *B. pseudomallei* strain HBPUB10073A | Present | Present |
|  | *B. pseudomallei* strain HBPUB10074A | Present | Present |
|  | *B. pseudomallei* strain HBPUB10084A | Present | Present |
|  | *B. pseudomallei* strain HBPUB10086A | Present | Present |
|  | *B. pseudomallei* strain HBPUB10083A | Present | Present |
|  | *B. pseudomallei* strain HBPUB10100A | Present | Present |
|  | *B. pseudomallei* strain HBPUB10096A | Present | Present |
|  | *B. pseudomallei* strain HBPUB10098B | Present | Present |
|  | *B. pseudomallei* strain HBPUB10093A | Present | Present |
|  | *B. pseudomallei* strain HBPUB10094B | Present | Present |
|  | *B. pseudomallei* strain HBPUB10101A | Present | Present |
|  | *B. pseudomallei* strain HBPUB10102A | Present | Present |
|  | *B. pseudomallei* strain HBPUB10105A | Present | Present |
|  | *B. pseudomallei* strain HBPUB10115A | Present | Present |
|  | *B. pseudomallei* strain HBPUB10120A | Present | Present |
|  | *B. pseudomallei* strain HBPUB10108A | Present | Present |
|  | *B. pseudomallei* strain HBPUB10119A | Present | Present |
|  | *B. pseudomallei* strain HBPUB10117A | Present | Present |
|  | *B. pseudomallei* strain HBPUB10114A | Present | Present |
|  | *B. pseudomallei* strain HBPUB10123A | Present | Present |
|  | *B. pseudomallei* strain HBPUB10122A | Present | Present |
|  | *B. pseudomallei* strain HBPUB10130B | Present | Present |
|  | *B. pseudomallei* strain HBPUB10132C | Present | Present |
|  | *B. pseudomallei* strain HBPUB10139A | Present | Present |
|  | *B. pseudomallei* strain HBPUB10135A | Present | Present |
|  | *B. pseudomallei* strain HBPUB10134A | Present | Present |
|  | *B. pseudomallei* strain HBPUB10136A | Present | Present |
|  | *B. pseudomallei* strain HBPUB10151A | Present | Present |
|  | *B. pseudomallei* strain HBPUB10144B | Present | Present |
|  | *B. pseudomallei* strain HBPUB10146A | Present | Present |
|  | *B. pseudomallei* strain HBPUB10152A | Present | Present |
|  | *B. pseudomallei* strain HBPUB10153B | Present | Present |
|  | *B. pseudomallei* strain HBPUB10140A | Present | Present |
|  | *B. pseudomallei* strain HBPUB10157A | Present | Present |
|  | *B. pseudomallei* strain HBPUB10158A | Present | Present |
|  | *B. pseudomallei* strain HBPUB10155C | Present | Present |
|  | *B. pseudomallei* strain HBPUB10156C | Present | Present |
|  | *B. pseudomallei* strain HBPUB10160A | Present | Present |
|  | *B. pseudomallei* strain HBPUB10164A | Present | Present |
|  | *B. pseudomallei* strain HBPUB10161A | Present | Present |
|  | *B. pseudomallei* strain HBPUB10163A | Present | Present |
|  | *B. pseudomallei* strain HBPUB10165A | Present | Present |
|  | *B. pseudomallei* strain HBPUB10171A | Present | Present |
|  | *B. pseudomallei* strain HBPUB10173B | Present | Present |
|  | *B. pseudomallei* strain HBPUB10177C | Present | Present |
|  | *B. pseudomallei* strain HBPUB10175C | Present | Present |
|  | *B. pseudomallei* strain HBPUB10191A | Present | Present |
|  | *B. pseudomallei* strain HBPUB10187A | Present | Present |
|  | *B. pseudomallei* strain HBPUB10186B | Present | Present |
|  | *B. pseudomallei* strain HBPUB10188A | Present | Present |
|  | *B. pseudomallei* strain HBPUB10184A | Present | Present |
|  | *B. pseudomallei* strain HBPUB10182A | Present | Present |
|  | *B. pseudomallei* strain HBPUB10183A | Present | Present |
|  | *B. pseudomallei* strain HBPUB10193A | Present | Present |
|  | *B. pseudomallei* strain HBPUB10192C | Present | Present |
|  | *B. pseudomallei* strain HbPUB10195A | Present | Present |
|  | *B. pseudomallei* strain HBPUB10194A | Present | Present |
|  | *B. pseudomallei* strain HBPUB10202A | Present | Present |
|  | *B. pseudomallei* strain HBPUB10198A | Present | Present |
|  | *B. pseudomallei* strain HBPUB10196A | Present | Present |
|  | *B. pseudomallei* strain HBPUB10199A | Present | Present |
|  | *B. pseudomallei* strain HBPUB10200A | Present | Present |
|  | *B. pseudomallei* strain HBPUB10206A | Present | Present |
|  | *B. pseudomallei* strain HBPUB10197A | Present | Present |
|  | *B. pseudomallei* strain HBPUB10201B | Present | Present |
|  | *B. pseudomallei* strain HBPUB10207A | Present | Present |
|  | *B. pseudomallei* strain HBPUB10208A | Present | Present |
|  | *B. pseudomallei* strain HBPUB10211A | Present | Present |
|  | *B. pseudomallei* strain HBPUB10212A | Present | Present |
|  | *B. pseudomallei* strain HBPUB10217B | Present | Present |
|  | *B. pseudomallei* strain HBPUB10216A | Present | Present |
|  | *B. pseudomallei* strain HBPUB10224A | Present | Present |
|  | *B. pseudomallei* strain HBPUB10213D | Present | Present |
|  | *B. pseudomallei* strain HBPUB10215B | Present | Present |
|  | *B. pseudomallei* strain HBPUB10221A | Present | Present |
|  | *B. pseudomallei* strain HBPUB10228B | Present | Present |
|  | *B. pseudomallei* strain HBPUB10229A | Present | Present |
|  | *B. pseudomallei* strain HBPUB10230D | Present | Present |
|  | *B. pseudomallei* strain HBPUB10234A | Present | Present |
|  | *B. pseudomallei* strain HBPUB10238C | Present | Present |
|  | *B. pseudomallei* strain HBPUB10239A | Present | Present |
|  | *B. pseudomallei* strain HBPUB10237B | Present | Present |
|  | *B. pseudomallei* strain HBPUB10242A | Present | Present |
|  | *B. pseudomallei* strain HBPUB10240A | Present | Present |
|  | *B. pseudomallei* strain HBPUB10246A | Present | Present |
|  | *B. pseudomallei* strain HBPUB10243A | Present | Present |
|  | *B. pseudomallei* strain HBPUB10244A | Present | Present |
|  | *B. pseudomallei* strain HBPUB10247A | Present | Present |
|  | *B. pseudomallei* strain HBPUB10248A | Present | Present |
|  | *B. pseudomallei* strain HBPUB10249B | Present | Present |
|  | *B. pseudomallei* strain HBPUB10260A | Present | Present |
|  | *B. pseudomallei* strain HBPUB10250A | Present | Present |
|  | *B. pseudomallei* strain HBPUB10255A | Present | Present |
|  | *B. pseudomallei* strain HBPUB10254C | Present | Present |
|  | *B. pseudomallei* strain HBPUB10269A | Present | Present |
|  | *B. pseudomallei* strain HBPUB10263A | Present | Present |
|  | *B. pseudomallei* strain HBPUB10265B | Present | Present |
|  | *B. pseudomallei* strain HBPUB10271A | Present | Present |
|  | *B. pseudomallei* strain HBPUB10273A | Present | Present |
|  | *B. pseudomallei* strain HBPUB10275C | Present | Present |
|  | *B. pseudomallei* strain HBPUB10279A | Present | Present |
|  | *B. pseudomallei* strain HBPUB10276A | Present | Present |
|  | *B. pseudomallei* strain HBPUB10281A | Present | Present |
|  | *B. pseudomallei* strain HBPUB10277A | Present | Present |
|  | *B. pseudomallei* strain HBPUB10302A | Present | Present |
|  | *B. pseudomallei* strain HBPUB10282C | Present | Present |
|  | *B. pseudomallei* strain HBPUB10283A | Present | Present |
|  | *B. pseudomallei* strain HBPUB10298A | Present | Present |
|  | *B. pseudomallei* strain HBPUB10299A | Present | Present |
|  | *B. pseudomallei* strain HBPUB10297A | Present | Present |
|  | *B. pseudomallei* strain HBPUB10303A | Present | Present |
|  | *B. pseudomallei* strain HBPUB10306A | Present | Present |
|  | *B. pseudomallei* strain HBPUB10304A | Present | Present |
|  | *B. pseudomallei* strain HBPUB10309A | Present | Present |
|  | *B. pseudomallei* strain HBPUB10308A | Present | Present |
|  | *B. pseudomallei* strain HBPUB10314A | Present | Present |
|  | *B. pseudomallei* strain HBPUB10307A | Present | Present |
|  | *B. pseudomallei* strain HBPUB10313B | Present | Present |
|  | *B. pseudomallei* strain HBPUB10311A | Present | Present |
|  | *B. pseudomallei* strain HBPUB10316A | Present | Present |
|  | *B. pseudomallei* strain HBPUB10315A | Present | Present |
|  | *B. pseudomallei* strain HBPUB10318B | Present | Present |
|  | *B. pseudomallei* strain HBPUB10319A | Present | Present |
|  | *B. pseudomallei* strain HBPUB10320A | Present | Present |
|  | *B. pseudomallei* strain HBPUB10323A | Present | Present |
|  | *B. pseudomallei* strain HBPUB10324A | Present | Present |
|  | *B. pseudomallei* strain HBPUB10325A | Present | Present |
|  | *B. pseudomallei* strain HBPUB10334A | Present | Present |
|  | *B. pseudomallei* strain HBPUB10331B | Present | Present |
|  | *B. pseudomallei* strain HBPUB10332A | Present | Present |
|  | *B. pseudomallei* strain HBPUB10333A | Present | Present |
|  | *B. pseudomallei* strain HBPUB10338A | Present | Present |
|  | *B. pseudomallei* strain HBPUB10336A | Present | Present |
|  | *B. pseudomallei* strain HBPUB10335A | Present | Present |
|  | *B. pseudomallei* strain HBPUB10326A | Present | Present |
|  | *B. pseudomallei* strain HBPUB10340B | Present | Present |
|  | *B. pseudomallei* strain HBPUB10341C | Present | Present |
|  | *B. pseudomallei* strain HBPUB10348A | Present | Present |
|  | *B. pseudomallei* strain HBPUB10342B | Present | Present |
|  | *B. pseudomallei* strain HBPUB10347B | Present | Present |
|  | *B. pseudomallei* strain HBPUB10346A | Present | Present |
|  | *B. pseudomallei* strain HBPUB10350A | Present | Present |
|  | *B. pseudomallei* strain HBPUB10351A | Present | Present |
|  | *B. pseudomallei* strain HBPUB10354A | Present | Present |
|  | *B. pseudomallei* strain HBPUB10353A | Present | Present |
|  | *B. pseudomallei* strain HBPUB10349A | Present | Present |
|  | *B. pseudomallei* strain HBPUB10355A | Present | Present |
|  | *B. pseudomallei* strain HBPUB10356A | Present | Present |
|  | *B. pseudomallei* strain HBPUB10358A | Present | Present |
|  | *B. pseudomallei* strain HBPUB10362A | Present | Present |
|  | *B. pseudomallei* strain HBPUB10360B | Present | Present |
|  | *B. pseudomallei* strain HBPUB10361A | Present | Present |
|  | *B. pseudomallei* strain HBPUB10366A | Present | Present |
|  | *B. pseudomallei* strain HBPUB10365A | Present | Present |
|  | *B. pseudomallei* strain HBPUB10369A | Present | Present |
|  | *B. pseudomallei* strain HBPUB10371B | Present | Present |
|  | *B. pseudomallei* strain HBPUB10370A | Present | Present |
|  | *B. pseudomallei* strain HBPUB10376C | Present | Present |
|  | *B. pseudomallei* strain HBPUB10373A | Present | Present |
|  | *B. pseudomallei* strain HBPUB10385A | Present | Present |
|  | *B. pseudomallei* strain HBPUB10378A | Present | Present |
|  | *B. pseudomallei* strain HBPUB10384A | Present | Present |
|  | *B. pseudomallei* strain HBPUB10381C | Present | Present |
|  | *B. pseudomallei* strain HBPUB10377A | Present | Present |
|  | *B. pseudomallei* strain HBPUB10386B | Present | Present |
|  | *B. pseudomallei* strain HBPUB10387A | Present | Present |
|  | *B. pseudomallei* strain HBPUB10389A | Present | Present |
|  | *B. pseudomallei* strain HBPUB10388A | Present | Present |
|  | *B. pseudomallei* strain HBPUB10392A | Present | Present |
|  | *B. pseudomallei* strain HBPUB10390A | Present | Present |
|  | *B. pseudomallei* strain HBPUB10398A | Present | Present |
|  | *B. pseudomallei* strain HBPUB10400A | Present | Present |
|  | *B. pseudomallei* strain HBPUB10398A-GS | Present | Present |
|  | *B. pseudomallei* strain HBPUB10395A | Present | Present |
|  | *B. pseudomallei* strain HBPUB10393A | Present | Present |
|  | *B. pseudomallei* strain HBPUB10403A | Present | Present |
|  | *B. pseudomallei* strain HBPUB10401A | Present | Present |
|  | *B. pseudomallei* strain HBPUB10402A | Present | Present |
|  | *B. pseudomallei* strain HBPUB10405A | Present | Present |
|  | *B. pseudomallei* strain HBPUB10406A | Present | Present |
|  | *B. pseudomallei* strain HBPUB10409A | Present | Present |
|  | *B. pseudomallei* strain HBPUB10410A | Present | Present |
|  | *B. pseudomallei* strain HBPUB10413B | Present | Present |
|  | *B. pseudomallei* strain HBPUB10411A | Present | Present |
|  | *B. pseudomallei* strain HBPUB10414A | Present | Present |
|  | *B. pseudomallei* strain HBPUB10416A | Present | Present |
|  | *B. pseudomallei* strain HBPUB10417A | Present | Present |
|  | *B. pseudomallei* strain HBPUB10415D | Present | Present |
|  | *B. pseudomallei* strain HBPUB10418A | Present | Present |
|  | *B. pseudomallei* strain HBPUB10420A | Present | Present |
|  | *B. pseudomallei* strain HBPUB10430A | Present | Present |
|  | *B. pseudomallei* strain HBPUB10431A | Present | Present |
|  | *B. pseudomallei* strain HBPUB10425B | Present | Present |
|  | *B. pseudomallei* strain HBPUB10423B | Present | Present |
|  | *B. pseudomallei* strain HBPUB10439A | Present | Present |
|  | *B. pseudomallei* strain HBPUB10436A | Present | Present |
|  | *B. pseudomallei* strain HBPUB10438B | Present | Present |
|  | *B. pseudomallei* strain HBPUB10432A | Present | Present |
|  | *B. pseudomallei* strain HBPUB10442A | Present | Present |
|  | *B. pseudomallei* strain HBPUB10444A | Present | Present |
|  | *B. pseudomallei* strain HBPUB10446C | Present | Present |
|  | *B. pseudomallei* strain HBPUB10448A | Present | Present |
|  | *B. pseudomallei* strain HBPUB10455A | Present | Present |
|  | *B. pseudomallei* strain HBPUB10449A | Present | Present |
|  | *B. pseudomallei* strain HBPUB10447A | Present | Present |
|  | *B. pseudomallei* strain HBPUB10451B | Present | Present |
|  | *B. pseudomallei* strain HBPUB10467B | Present | Present |
|  | *B. pseudomallei* strain HBPUB10461A | Present | Present |
|  | *B. pseudomallei* strain HBPUB10460A | Present | Present |
|  | *B. pseudomallei* strain HBPUB10465A | Present | Present |
|  | *B. pseudomallei* strain HBPUB10469A | Present | Present |
|  | *B. pseudomallei* strain HBPUB10480A | Present | Present |
|  | *B. pseudomallei* strain HBPUB10476B | Present | Present |
|  | *B. pseudomallei* strain HBPUB10483A | Present | Present |
|  | *B. pseudomallei* strain HBPUB10501A | Present | Present |
|  | *B. pseudomallei* strain HBPUB10497A | Present | Present |
|  | *B. pseudomallei* strain HBPUB10488A | Present | Present |
|  | *B. pseudomallei* strain HBPUB10515A | Present | Present |
|  | *B. pseudomallei* strain HBPUB10522A | Present | Present |
|  | *B. pseudomallei* strain HBPUB10529A | Present | Present |
|  | *B. pseudomallei* strain HBPUB10532A | Present | Present |
|  | *B. pseudomallei* strain HBPUB10534C | Present | Present |
|  | *B. pseudomallei* strain HBPUB10542A | Present | Present |
|  | *B. pseudomallei* strain HBPUB10544B | Present | Present |
|  | *B. pseudomallei* strain HBPUB10548A | Present | Present |
|  | *B. pseudomallei* strain HBPUB10546A | Present | Present |
|  | *B. pseudomallei* strain HBPUB10549A | Present | Present |
|  | *B. pseudomallei* strain HBPUB10554A | Present | Present |
|  | *B. pseudomallei* strain HBPUB10550A | Present | Present |
|  | *B. pseudomallei* strain HBPUB10557A | Present | Present |
|  | *B. pseudomallei* strain HBPUB10556B | Present | Present |
|  | *B. pseudomallei* strain HBPUB10560A | Present | Present |
|  | *B. pseudomallei* strain HBPUB10564A | Present | Present |
|  | *B. pseudomallei* strain HBPUB10566C | Present | Present |
|  | *B. pseudomallei* strain HBPUB10571A | Present | Present |
|  | *B. pseudomallei* strain HBPUB10574A | Present | Present |
|  | *B. pseudomallei* strain HBPUB10577A | Present | Present |
|  | *B. pseudomallei* strain HBPUB10575B | Present | Present |
|  | *B. pseudomallei* strain HBPUB10581A | Present | Present |
|  | *B. pseudomallei* strain HBPUB10584A | Present | Present |
|  | *B. pseudomallei* strain HBPUB10587C | Present | Present |
|  | *B. pseudomallei* strain HBPUB10596A | Present | Present |
|  | *B. pseudomallei* strain HBPUB10591B | Present | Present |
|  | *B. pseudomallei* strain HBPUB10598A | Present | Present |
|  | *B. pseudomallei* strain HBPUB10602C | Present | Present |
|  | *B. pseudomallei* strain HBPUB10601A | Present | Present |
|  | *B. pseudomallei* strain HBPUB10604A | Present | Present |
|  | *B. pseudomallei* strain HBPUB10609A | Present | Present |
|  | *B. pseudomallei* strain HBPUB10603A | Present | Present |
|  | *B. pseudomallei* strain HBPUB10629B | Present | Present |
|  | *B. pseudomallei* strain HBPUB10625A | Present | Present |
|  | *B. pseudomallei* strain HBPUB10618A | Present | Present |
|  | *B. pseudomallei* strain HBPUB10623A | Present | Present |
|  | *B. pseudomallei* strain HBPUB10633A | Present | Present |
|  | *B. pseudomallei* strain HBPUB10630A | Present | Present |
|  | *B. pseudomallei* strain HBPUB10640A | Present | Present |
|  | *B. pseudomallei* strain HBPUB10642A | Present | Present |
|  | *B. pseudomallei* strain HBPUB10643A | Present | Present |
|  | *B. pseudomallei* strain HBPUB10645A | Present | Present |
|  | *B. pseudomallei* strain HBPUB10649A | Present | Present |
|  | *B. pseudomallei* strain HBPUB10658A | Present | Present |
|  | *B. pseudomallei* strain HBPUB10661B | Present | Present |
|  | *B. pseudomallei* strain HBPUB10650A | Present | Present |
|  | *B. pseudomallei* strain HBPUB10657A | Present | Present |
|  | *B. pseudomallei* strain HBPUB10652C | Present | Present |
|  | *B. pseudomallei* strain HBPUB10668A | Present | Present |
|  | *B. pseudomallei* strain HBPUB10669A | Present | Present |
|  | *B. pseudomallei* strain HBPUB10670B | Present | Present |
|  | *B. pseudomallei* strain HBPUB10671A | Present | Present |
|  | *B. pseudomallei* strain HBPUB10679A | Present | Present |
|  | *B. pseudomallei* strain HBPUB10687A | Present | Present |
|  | *B. pseudomallei* strain HBPUB10685A | Present | Present |
|  | *B. pseudomallei* strain HBPUB10688A | Present | Present |
|  | *B. pseudomallei* strain HBPUB10681A | Present | Present |
|  | *B. pseudomallei* strain HBPUB5603A | Present | Present |
|  | *B. pseudomallei* strain A-041-05-2-02 | Present | Present |
|  | *B. pseudomallei* strain A-041-05-2-01 | Present | Present |
|  | *B. pseudomallei* strain A-041-05-2-04 | Present | Present |
|  | *B. pseudomallei* strain A-041-05-2-03 | Present | Present |
|  | *B. pseudomallei* strain A-041-05-2-05 | Present | Present |
|  | *B. pseudomallei* strain A-041-05-2-07 | Present | Present |
|  | *B. pseudomallei* strain A-041-05-2-06 | Present | Present |
|  | *B. pseudomallei* strain A-041-05-2-08 | Present | Present |
|  | *B. pseudomallei* strain A-041-05-2-10 | Present | Present |
|  | *B. pseudomallei* strain A-050-05-2-01 | Present | Present |
|  | *B. pseudomallei* strain A-050-05-2-02 | Present | Present |
|  | *B. pseudomallei* strain A-041-05-2-09 | Present | Present |
|  | *B. pseudomallei* strain A-050-05-2-05 | Present | Present |
|  | *B. pseudomallei* strain A-050-05-2-04 | Present | Present |
|  | *B. pseudomallei* strain A-050-05-2-03 | Present | Present |
|  | *B. pseudomallei* strain A-050-05-2-06 | Present | Present |
|  | *B. pseudomallei* strain A-050-05-2-07 | Present | Present |
|  | *B. pseudomallei* strain A-050-05-2-10 | Present | Present |
|  | *B. pseudomallei* strain A-050-05-2-09 | Present | Present |
|  | *B. pseudomallei* strain A-055-05-1-01 | Present | Present |
|  | *B. pseudomallei* strain A-052-05-1-01 | Present | Present |
|  | *B. pseudomallei* strain A-050-05-2-08 | Present | Present |
|  | *B. pseudomallei* strain A-055-05-1-06 | Present | Present |
|  | *B. pseudomallei* strain A-055-05-1-04 | Present | Present |
|  | *B. pseudomallei* strain A-055-05-1-05 | Present | Present |
|  | *B. pseudomallei* strain A-055-05-1-03 | Present | Present |
|  | *B. pseudomallei* strain A-055-05-1-02 | Present | Present |
|  | *B. pseudomallei* strain A-055-05-1-07 | Present | Present |
|  | *B. pseudomallei* strain A-055-05-1-09 | Present | Present |
|  | *B. pseudomallei* strain A-055-05-1-08 | Present | Present |
|  | *B. pseudomallei* strain A-058-02-2-01 | Present | Present |
|  | *B. pseudomallei* strain A-055-05-1-10 | Present | Present |
|  | *B. pseudomallei* strain A-058-02-2-04 | Present | Present |
|  | *B. pseudomallei* strain A-058-02-2-07 | Present | Present |
|  | *B. pseudomallei* strain A-058-02-2-05 | Present | Present |
|  | *B. pseudomallei* strain A-058-02-2-02 | Present | Present |
|  | *B. pseudomallei* strain A-058-02-2-06 | Present | Present |
|  | *B. pseudomallei* strain A-058-02-2-08 | Present | Present |
|  | *B. pseudomallei* strain A-058-02-2-09 | Present | Present |
|  | *B. pseudomallei* strain A-064-05-2-01 | Present | Present |
|  | *B. pseudomallei* strain A-058-02-2-10 | Present | Present |
|  | *B. pseudomallei* strain A-064-05-2-02 | Present | Present |
|  | *B. pseudomallei* strain A-064-05-2-03 | Present | Present |
|  | *B. pseudomallei* strain A-064-05-2-05 | Present | Present |
|  | *B. pseudomallei* strain A-064-05-2-04 | Present | Present |
|  | *B. pseudomallei* strain A-064-05-2-06 | Present | Present |
|  | *B. pseudomallei* strain A-064-05-2-07 | Present | Present |
|  | *B. pseudomallei* strain A-064-05-2-08 | Present | Present |
|  | *B. pseudomallei* strain A-064-05-2-10 | Present | Present |
|  | *B. pseudomallei* strain A-064-05-2-09 | Present | Present |
|  | *B. pseudomallei* strain A-095-05-2-01 | Present | Present |
|  | *B. pseudomallei* strain A-095-05-2-02 | Present | Present |
|  | *B. pseudomallei* strain A-095-05-2-03 | Present | Present |
|  | *B. pseudomallei* strain A-095-05-2-07 | Present | Present |
|  | *B. pseudomallei* strain A-095-05-2-08 | Present | Present |
|  | *B. pseudomallei* strain A-095-05-2-05 | Present | Present |
|  | *B. pseudomallei* strain A-095-05-2-04 | Present | Present |
|  | *B. pseudomallei* strain A-095-05-2-06 | Present | Present |
|  | *B. pseudomallei* strain A-095-05-2-10 | Present | Present |
|  | *B. pseudomallei* strain A-095-05-2-09 | Present | Present |
|  | *B. pseudomallei* strain A-099-05-1-01 | Present | Present |
|  | *B. pseudomallei* strain A-099-05-1-03 | Present | Present |
|  | *B. pseudomallei* strain A-099-05-1-02 | Present | Present |
|  | *B. pseudomallei* strain A-099-05-1-04 | Present | Present |
|  | *B. pseudomallei* strain A-099-05-2-07 | Present | Present |
|  | *B. pseudomallei* strain A-099-05-2-09 | Present | Present |
|  | *B. pseudomallei* strain A-099-05-2-08 | Present | Present |
|  | *B. pseudomallei* strain A-099-05-2-06 | Present | Present |
|  | *B. pseudomallei* strain A-099-05-2-05 | Present | Present |
|  | *B. pseudomallei* strain A-107-05-2-02 | Present | Present |
|  | *B. pseudomallei* strain A-099-05-2-10 | Present | Present |
|  | *B. pseudomallei* strain A-107-05-1-01 | Present | Present |
|  | *B. pseudomallei* strain A-107-05-2-03 | Present | Present |
|  | *B. pseudomallei* strain A-107-05-2-04 | Present | Present |
|  | *B. pseudomallei* strain A-107-05-2-05 | Present | Present |
|  | *B. pseudomallei* strain A-107-05-2-06 | Present | Present |
|  | *B. pseudomallei* strain A-107-05-2-07 | Present | Present |
|  | *B. pseudomallei* strain A-107-05-2-09 | Present | Present |
|  | *B. pseudomallei* strain A-107-05-2-08 | Present | Present |
|  | *B. pseudomallei* strain A-107-05-2-10 | Present | Present |
|  | *B. pseudomallei* strain A-161-02-1-04 | Present | Present |
|  | *B. pseudomallei* strain A-161-02-1-01 | Present | Present |
|  | *B. pseudomallei* strain A-161-02-1-03 | Present | Present |
|  | *B. pseudomallei* strain A-161-02-1-02 | Present | Present |
|  | *B. pseudomallei* strain A-161-02-1-05 | Present | Present |
|  | *B. pseudomallei* strain A-161-02-1-06 | Present | Present |
|  | *B. pseudomallei* strain A-161-02-1-08 | Present | Present |
|  | *B. pseudomallei* strain A-161-02-1-07 | Present | Present |
|  | *B. pseudomallei* strain A-161-02-1-09 | Present | Present |
|  | *B. pseudomallei* strain A-175-05-2-03 | Present | Present |
|  | *B. pseudomallei* strain A-175-05-2-02 | Present | Present |
|  | *B. pseudomallei* strain A-161-02-1-10 | Present | Present |
|  | *B. pseudomallei* strain A-175-05-2-05 | Present | Present |
|  | *B. pseudomallei* strain A-175-05-2-01 | Present | Present |
|  | *B. pseudomallei* strain A-175-05-2-06 | Present | Present |
|  | *B. pseudomallei* strain A-175-05-2-08 | Present | Present |
|  | *B. pseudomallei* strain A-175-05-2-10 | Present | Present |
|  | *B. pseudomallei* strain A-175-05-2-09 | Present | Present |
|  | *B. pseudomallei* strain A-177-01-2-02 | Present | Present |
|  | *B. pseudomallei* strain A-177-01-1-01 | Present | Present |
|  | *B. pseudomallei* strain A-177-01-2-07 | Present | Present |
|  | *B. pseudomallei* strain A-177-01-2-06 | Present | Present |
|  | *B. pseudomallei* strain A-177-01-2-04 | Present | Present |
|  | *B. pseudomallei* strain A-177-01-2-03 | Present | Present |
|  | *B. pseudomallei* strain A-177-01-2-05 | Present | Present |
|  | *B. pseudomallei* strain A-177-01-2-09 | Present | Present |
|  | *B. pseudomallei* strain A-177-01-2-10 | Present | Present |
|  | *B. pseudomallei* strain A-177-01-2-08 | Present | Present |
|  | *B. pseudomallei* strain A-202-02-1-01 | Present | Present |
|  | *B. pseudomallei* strain A-202-02-1-02 | Present | Present |
|  | *B. pseudomallei* strain A-202-02-1-03 | Present | Present |
|  | *B. pseudomallei* strain A-202-02-1-08 | Present | Present |
|  | *B. pseudomallei* strain A-202-02-1-07 | Present | Present |
|  | *B. pseudomallei* strain A-202-02-1-06 | Present | Present |
|  | *B. pseudomallei* strain A-202-02-1-04 | Present | Present |
|  | *B. pseudomallei* strain A-202-02-1-05 | Present | Present |
|  | *B. pseudomallei* strain A-229-05-2-01 | Present | Present |
|  | *B. pseudomallei* strain A-229-05-2-02 | Present | Present |
|  | *B. pseudomallei* strain A-202-02-1-09 | Present | Present |
|  | *B. pseudomallei* strain A-202-02-1-10 | Present | Present |
|  | *B. pseudomallei* strain A-229-05-2-03 | Present | Present |
|  | *B. pseudomallei* strain A-229-05-2-05 | Present | Present |
|  | *B. pseudomallei* strain A-229-05-2-04 | Present | Present |
|  | *B. pseudomallei* strain A-229-05-2-07 | Present | Present |
|  | *B. pseudomallei* strain A-229-05-2-08 | Present | Present |
|  | *B. pseudomallei* strain A-229-05-2-06 | Present | Present |
|  | *B. pseudomallei* strain A-266-02-1-03 | Present | Present |
|  | *B. pseudomallei* strain A-229-05-2-10 | Present | Present |
|  | *B. pseudomallei* strain A-266-02-1-01 | Present | Present |
|  | *B. pseudomallei* strain A-229-05-2-09 | Present | Present |
|  | *B. pseudomallei* strain A-266-02-1-02 | Present | Present |
|  | *B. pseudomallei* strain A-266-02-1-04 | Present | Present |
|  | *B. pseudomallei* strain A-266-02-1-05 | Present | Present |
|  | *B. pseudomallei* strain A-266-02-1-08 | Present | Present |
|  | *B. pseudomallei* strain A-266-02-1-06 | Present | Present |
|  | *B. pseudomallei* strain A-266-02-1-07 | Present | Present |
|  | *B. pseudomallei* strain A-266-02-1-09 | Present | Present |
|  | *B. pseudomallei* strain A-277-02-2-02 | Present | Present |
|  | *B. pseudomallei* strain A-266-02-1-10 | Present | Present |
|  | *B. pseudomallei* strain A-277-02-2-03 | Present | Present |
|  | *B. pseudomallei* strain A-277-02-2-01 | Present | Present |
|  | *B. pseudomallei* strain A-277-02-2-04 | Present | Present |
|  | *B. pseudomallei* strain A-277-02-2-06 | Present | Present |
|  | *B. pseudomallei* strain A-277-02-2-05 | Present | Present |
|  | *B. pseudomallei* strain A-277-02-2-07 | Present | Present |
|  | *B. pseudomallei* strain A-277-02-2-10 | Present | Present |
|  | *B. pseudomallei* strain A-278-05-2-03 | Present | Present |
|  | *B. pseudomallei* strain A-278-05-2-02 | Present | Present |
|  | *B. pseudomallei* strain A-277-02-2-09 | Present | Present |
|  | *B. pseudomallei* strain A-277-02-2-08 | Present | Present |
|  | *B. pseudomallei* strain A-278-05-2-05 | Present | Present |
|  | *B. pseudomallei* strain A-278-05-2-04 | Present | Present |
|  | *B. pseudomallei* strain A-278-05-2-06 | Present | Present |
|  | *B. pseudomallei* strain A-278-05-2-07 | Present | Present |
|  | *B. pseudomallei* strain A-278-05-2-09 | Present | Present |
|  | *B. pseudomallei* strain A-281-02-2-03 | Present | Present |
|  | *B. pseudomallei* strain A-278-05-2-10 | Present | Present |
|  | *B. pseudomallei* strain A-281-02-2-02 | Present | Present |
|  | *B. pseudomallei* strain A-281-02-2-01 | Present | Present |
|  | *B. pseudomallei* strain A-278-05-2-08 | Present | Present |
|  | *B. pseudomallei* strain A-281-02-2-06 | Present | Present |
|  | *B. pseudomallei* strain A-281-02-2-07 | Present | Present |
|  | *B. pseudomallei* strain A-281-02-2-05 | Present | Present |
|  | *B. pseudomallei* strain A-281-02-2-04 | Present | Present |
|  | *B. pseudomallei* strain A-281-02-2-08 | Present | Present |
|  | *B. pseudomallei* strain A-282-02-1-04 | Present | Present |
|  | *B. pseudomallei* strain A-281-02-2-10 | Present | Present |
|  | *B. pseudomallei* strain A-288-02-1-02 | Present | Present |
|  | *B. pseudomallei* strain A-288-02-1-01 | Present | Present |
|  | *B. pseudomallei* strain A-281-02-2-09 | Present | Present |
|  | *B. pseudomallei* strain A-288-02-1-05 | Present | Present |
|  | *B. pseudomallei* strain A-288-02-1-04 | Present | Present |
|  | *B. pseudomallei* strain A-288-02-1-06 | Present | Present |
|  | *B. pseudomallei* strain A-288-02-1-03 | Present | Present |
|  | *B. pseudomallei* strain A-288-02-1-07 | Present | Present |
|  | *B. pseudomallei* strain A-288-02-1-08 | Present | Present |
|  | *B. pseudomallei* strain A-288-02-1-10 | Present | Present |
|  | *B. pseudomallei* strain A-288-02-1-09 | Present | Present |
|  | *B. pseudomallei* strain A-294-05-2-02 | Present | Present |
|  | *B. pseudomallei* strain A-294-05-2-01 | Present | Present |
|  | *B. pseudomallei* strain A-294-05-2-06 | Present | Present |
|  | *B. pseudomallei* strain A-294-05-2-07 | Present | Present |
|  | *B. pseudomallei* strain A-294-05-2-05 | Present | Present |
|  | *B. pseudomallei* strain A-294-05-2-03 | Present | Present |
|  | *B. pseudomallei* strain A-294-05-2-04 | Present | Present |
|  | *B. pseudomallei* strain A-294-05-2-08 | Present | Present |
|  | *B. pseudomallei* strain A-294-05-2-09 | Present | Present |
|  | *B. pseudomallei* strain A-294-05-2-10 | Present | Present |
|  | *B. pseudomallei* strain A-297-05-2-01 | Present | Present |
|  | *B. pseudomallei* strain A-297-05-2-02 | Present | Present |
|  | *B. pseudomallei* strain A-297-05-2-07 | Present | Present |
|  | *B. pseudomallei* strain A-297-05-2-04 | Present | Present |
|  | *B. pseudomallei* strain A-297-05-2-06 | Present | Present |
|  | *B. pseudomallei* strain A-297-05-2-03 | Present | Present |
|  | *B. pseudomallei* strain A-297-05-2-08 | Present | Present |
|  | *B. pseudomallei* strain A-297-05-2-05 | Present | Present |
|  | *B. pseudomallei* strain A-297-05-2-09 | Present | Present |
|  | *B. pseudomallei* strain A-297-05-2-10 | Present | Present |
|  | *B. pseudomallei* strain A-305-02-1-01 | Present | Present |
|  | *B. pseudomallei* strain A-305-02-1-02 | Present | Present |
|  | *B. pseudomallei* strain A-305-02-1-04 | Present | Present |
|  | *B. pseudomallei* strain A-305-02-1-07 | Present | Present |
|  | *B. pseudomallei* strain A-305-02-1-05 | Present | Present |
|  | *B. pseudomallei* strain A-305-02-1-08 | Present | Present |
|  | *B. pseudomallei* strain A-305-02-1-06 | Present | Present |
|  | *B. pseudomallei* strain A-305-02-1-09 | Present | Present |
|  | *B. pseudomallei* strain A-305-02-1-10 | Present | Present |
|  | *B. pseudomallei* strain A-308-01-1-02 | Present | Present |
|  | *B. pseudomallei* strain A-308-01-1-03 | Present | Present |
|  | *B. pseudomallei* strain A-308-01-1-04 | Present | Present |
|  | *B. pseudomallei* strain A-308-01-1-07 | Present | Present |
|  | *B. pseudomallei* strain A-308-01-1-09 | Present | Present |
|  | *B. pseudomallei* strain A-308-01-1-08 | Present | Present |
|  | *B. pseudomallei* strain A-308-01-1-06 | Present | Present |
|  | *B. pseudomallei* strain A-308-01-1-10 | Present | Present |
|  | *B. pseudomallei* strain A-328-05-1-01 | Present | Present |
|  | *B. pseudomallei* strain A-328-05-1-05 | Present | Present |
|  | *B. pseudomallei* strain A-328-05-1-03 | Present | Present |
|  | *B. pseudomallei* strain A-328-05-1-04 | Present | Present |
|  | *B. pseudomallei* strain A-328-05-1-02 | Present | Present |
|  | *B. pseudomallei* strain A-328-05-1-06 | Present | Present |
|  | *B. pseudomallei* strain A-328-05-1-08 | Present | Present |
|  | *B. pseudomallei* strain A-328-05-1-09 | Present | Present |
|  | *B. pseudomallei* strain A-328-05-1-07 | Present | Present |
|  | *B. pseudomallei* strain A-329-05-2-01 | Present | Present |
|  | *B. pseudomallei* strain A-328-05-1-10 | Present | Present |
|  | *B. pseudomallei* strain A-329-05-2-03 | Present | Present |
|  | *B. pseudomallei* strain A-329-05-2-02 | Present | Present |
|  | *B. pseudomallei* strain A-329-05-2-06 | Present | Present |
|  | *B. pseudomallei* strain A-329-05-2-05 | Present | Present |
|  | *B. pseudomallei* strain A-329-05-2-09 | Present | Present |
|  | *B. pseudomallei* strain A-329-05-2-08 | Present | Present |
|  | *B. pseudomallei* strain A-329-05-2-07 | Present | Present |
|  | *B. pseudomallei* strain A-329-05-2-10 | Present | Present |
|  | *B. pseudomallei* strain A-330-05-1-03 | Present | Present |
|  | *B. pseudomallei* strain A-330-05-2-06 | Present | Present |
|  | *B. pseudomallei* strain A-330-05-1-02 | Present | Present |
|  | *B. pseudomallei* strain A-330-05-2-05 | Present | Present |
|  | *B. pseudomallei* strain A-330-05-1-01 | Present | Present |
|  | *B. pseudomallei* strain A-330-05-2-10 | Present | Present |
|  | *B. pseudomallei* strain A-330-05-2-07 | Present | Present |
|  | *B. pseudomallei* strain B-065-05-1-01 | Present | Present |
|  | *B. pseudomallei* strain A-330-05-2-08 | Present | Present |
|  | *B. pseudomallei* strain B-065-05-1-02 | Present | Present |
|  | *B. pseudomallei* strain B-065-05-1-06 | Present | Present |
|  | *B. pseudomallei* strain B-065-05-1-05 | Present | Present |
|  | *B. pseudomallei* strain B-065-05-1-04 | Present | Present |
|  | *B. pseudomallei* strain B-065-05-1-03 | Present | Present |
|  | *B. pseudomallei* strain B-163-05-2-03 | Present | Present |
|  | *B. pseudomallei* strain B-163-05-2-05 | Present | Present |
|  | *B. pseudomallei* strain B-163-05-2-02 | Present | Present |
|  | *B. pseudomallei* strain B-163-05-2-06 | Present | Present |
|  | *B. pseudomallei* strain B-163-05-2-04 | Present | Present |
|  | *B. pseudomallei* strain B-163-05-2-01 | Present | Present |
|  | *B. pseudomallei* strain B-163-05-2-08 | Present | Present |
|  | *B. pseudomallei* strain B-180-05-1-01 | Present | Present |
|  | *B. pseudomallei* strain B-163-05-2-10 | Present | Present |
|  | *B. pseudomallei* strain B-163-05-2-09 | Present | Present |
|  | *B. pseudomallei* strain B-180-05-2-07 | Present | Present |
|  | *B. pseudomallei* strain B-180-05-2-06 | Present | Present |
|  | *B. pseudomallei* strain B-180-05-2-02 | Present | Present |
|  | *B. pseudomallei* strain B-180-05-2-03 | Present | Present |
|  | *B. pseudomallei* strain B-180-05-2-04 | Present | Present |
|  | *B. pseudomallei* strain B-180-05-2-05 | Present | Present |
|  | *B. pseudomallei* strain B-180-05-2-08 | Present | Present |
|  | *B. pseudomallei* strain B-180-05-2-10 | Present | Present |
|  | *B. pseudomallei* strain B-180-05-2-09 | Present | Present |
|  | *B. pseudomallei* strain B-182-05-2-01 | Present | Present |
|  | *B. pseudomallei* strain B-182-05-2-07 | Present | Present |
|  | *B. pseudomallei* strain B-182-05-2-04 | Present | Present |
|  | *B. pseudomallei* strain B-182-05-2-02 | Present | Present |
|  | *B. pseudomallei* strain B-182-05-2-06 | Present | Present |
|  | *B. pseudomallei* strain B-182-05-2-03 | Present | Present |
|  | *B. pseudomallei* strain B-182-05-2-05 | Present | Present |
|  | *B. pseudomallei* strain B-182-05-2-08 | Present | Present |
|  | *B. pseudomallei* strain B-182-05-2-09 | Present | Present |
|  | *B. pseudomallei* strain B-182-05-2-10 | Present | Present |
|  | *B. pseudomallei* strain B-227-02-2-01 | Present | Present |
|  | *B. pseudomallei* strain B-255-05-2-04 | Present | Present |
|  | *B. pseudomallei* strain B-255-05-2-06 | Present | Present |
|  | *B. pseudomallei* strain B-255-05-2-05 | Present | Present |
|  | *B. pseudomallei* strain B-255-05-2-03 | Present | Present |
|  | *B. pseudomallei* strain B-255-05-2-01 | Present | Present |
|  | *B. pseudomallei* strain B-255-05-2-02 | Present | Present |
|  | *B. pseudomallei* strain B-255-05-2-07 | Present | Present |
|  | *B. pseudomallei* strain B-255-05-2-08 | Present | Present |
|  | *B. pseudomallei* strain B-255-05-2-10 | Present | Present |
|  | *B. pseudomallei* strain B-286-02-1-01 | Present | Present |
|  | *B. pseudomallei* strain B-286-02-1-03 | Present | Present |
|  | *B. pseudomallei* strain B-286-02-1-06 | Present | Present |
|  | *B. pseudomallei* strain B-286-02-1-04 | Present | Present |
|  | *B. pseudomallei* strain B-286-02-1-05 | Present | Present |
|  | *B. pseudomallei* strain B-286-02-1-02 | Present | Present |
|  | *B. pseudomallei* strain B-286-02-1-07 | Present | Present |
|  | *B. pseudomallei* strain B-286-02-1-10 | Present | Present |
|  | *B. pseudomallei* strain B-286-02-1-08 | Present | Present |
|  | *B. pseudomallei* strain B-286-02-1-09 | Present | Present |
|  | *B. pseudomallei* strain B-296-02-1-01 | Present | Present |
|  | *B. pseudomallei* strain B-296-02-1-02 | Present | Present |
|  | *B. pseudomallei* strain B-296-02-1-03 | Present | Present |
|  | *B. pseudomallei* strain B-296-02-1-05 | Present | Present |
|  | *B. pseudomallei* strain B-296-02-1-06 | Present | Present |
|  | *B. pseudomallei* strain B-296-02-1-04 | Present | Present |
|  | *B. pseudomallei* strain B-296-02-1-08 | Present | Present |
|  | *B. pseudomallei* strain B-305-05-1-01 | Present | Present |
|  | *B. pseudomallei* strain B-296-02-1-10 | Present | Present |
|  | *B. pseudomallei* strain B-296-02-1-07 | Present | Present |
|  | *B. pseudomallei* strain B-296-02-1-09 | Present | Present |
|  | *B. pseudomallei* strain B-305-05-1-02 | Present | Present |
|  | *B. pseudomallei* strain B-305-05-1-04 | Present | Present |
|  | *B. pseudomallei* strain B-305-05-1-05 | Present | Present |
|  | *B. pseudomallei* strain B-305-05-1-03 | Present | Present |
|  | *B. pseudomallei* strain B-305-05-1-06 | Present | Present |
|  | *B. pseudomallei* strain B-305-05-1-07 | Present | Present |
|  | *B. pseudomallei* strain B-321-05-2-03 | Present | Present |
|  | *B. pseudomallei* strain B-321-05-2-02 | Present | Present |
|  | *B. pseudomallei* strain B-305-05-1-09 | Present | Present |
|  | *B. pseudomallei* strain B-305-05-1-08 | Present | Present |
|  | *B. pseudomallei* strain B-321-05-2-01 | Present | Present |
|  | *B. pseudomallei* strain B-321-05-2-04 | Present | Present |
|  | *B. pseudomallei* strain B-321-05-2-05 | Present | Present |
|  | *B. pseudomallei* strain B-321-05-2-06 | Present | Present |
|  | *B. pseudomallei* strain B-321-05-2-08 | Present | Present |
|  | *B. pseudomallei* strain B-321-05-2-09 | Present | Present |
|  | *B. pseudomallei* strain B-321-05-2-07 | Present | Present |
|  | *B. pseudomallei* strain B-321-05-2-10 | Present | Present |
|  | *B. pseudomallei* strain C-024-05-2-03 | Present | Present |
|  | *B. pseudomallei* strain C-024-05-2-01 | Present | Present |
|  | *B. pseudomallei* strain C-024-05-2-02 | Present | Present |
|  | *B. pseudomallei* strain C-024-05-2-05 | Present | Present |
|  | *B. pseudomallei* strain C-024-05-2-06 | Present | Present |
|  | *B. pseudomallei* strain C-024-05-2-04 | Present | Present |
|  | *B. pseudomallei* strain C-024-05-2-08 | Present | Present |
|  | *B. pseudomallei* strain C-024-05-2-07 | Present | Present |
|  | *B. pseudomallei* strain C-024-05-2-09 | Present | Present |
|  | *B. pseudomallei* strain C-024-05-2-10 | Present | Present |
|  | *B. pseudomallei* strain C-070-05-2-02 | Present | Present |
|  | *B. pseudomallei* strain C-070-05-2-01 | Present | Present |
|  | *B. pseudomallei* strain C-070-05-2-03 | Present | Present |
|  | *B. pseudomallei* strain C-070-05-2-06 | Present | Present |
|  | *B. pseudomallei* strain C-070-05-2-05 | Present | Present |
|  | *B. pseudomallei* strain C-070-05-2-04 | Present | Present |
|  | *B. pseudomallei* strain C-070-05-2-07 | Present | Present |
|  | *B. pseudomallei* strain C-070-05-2-08 | Present | Present |
|  | *B. pseudomallei* strain C-070-05-2-09 | Present | Present |
|  | *B. pseudomallei* strain C-070-05-2-10 | Present | Present |
|  | *B. pseudomallei* strain C-091-05-2-01 | Present | Present |
|  | *B. pseudomallei* strain C-091-05-2-05 | Present | Present |
|  | *B. pseudomallei* strain C-091-05-2-03 | Present | Present |
|  | *B. pseudomallei* strain C-091-05-2-04 | Present | Present |
|  | *B. pseudomallei* strain C-091-05-2-06 | Present | Present |
|  | *B. pseudomallei* strain C-091-05-2-02 | Present | Present |
|  | *B. pseudomallei* strain C-091-05-2-08 | Present | Present |
|  | *B. pseudomallei* strain C-091-05-2-09 | Present | Present |
|  | *B. pseudomallei* strain C-091-05-2-07 | Present | Present |
|  | *B. pseudomallei* strain C-091-05-2-10 | Present | Present |
|  | *B. pseudomallei* strain C-137-02-2-03 | Present | Present |
|  | *B. pseudomallei* strain C-137-02-2-05 | Present | Present |
|  | *B. pseudomallei* strain C-137-02-2-06 | Present | Present |
|  | *B. pseudomallei* strain C-137-02-2-01 | Present | Present |
|  | *B. pseudomallei* strain C-137-02-2-07 | Present | Present |
|  | *B. pseudomallei* strain C-137-02-2-04 | Present | Present |
|  | *B. pseudomallei* strain C-137-02-2-02 | Present | Present |
|  | *B. pseudomallei* strain C-137-02-2-10 | Present | Present |
|  | *B. pseudomallei* strain C-137-02-2-08 | Present | Present |
|  | *B. pseudomallei* strain C-137-02-2-09 | Present | Present |
|  | *B. pseudomallei* strain C-174-05-2-01 | Present | Present |
|  | *B. pseudomallei* strain C-174-05-2-04 | Present | Present |
|  | *B. pseudomallei* strain C-174-05-2-05 | Present | Present |
|  | *B. pseudomallei* strain C-174-05-2-02 | Present | Present |
|  | *B. pseudomallei* strain C-174-05-2-03 | Present | Present |
|  | *B. pseudomallei* strain C-174-05-2-09 | Present | Present |
|  | *B. pseudomallei* strain C-174-05-2-06 | Present | Present |
|  | *B. pseudomallei* strain C-174-05-2-10 | Present | Present |
|  | *B. pseudomallei* strain C-174-05-2-07 | Present | Present |
|  | *B. pseudomallei* strain C-174-05-2-08 | Present | Present |
|  | *B. pseudomallei* strain C-240-05-2-01 | Present | Present |
|  | *B. pseudomallei* strain C-175-05-1-01 | Present | Present |
|  | *B. pseudomallei* strain C-240-05-2-02 | Present | Present |
|  | *B. pseudomallei* strain C-240-05-2-03 | Present | Present |
|  | *B. pseudomallei* strain C-175-05-1-02 | Present | Present |
|  | *B. pseudomallei* strain C-240-05-2-04 | Present | Present |
|  | *B. pseudomallei* strain C-240-05-2-05 | Present | Present |
|  | *B. pseudomallei* strain C-240-05-2-06 | Present | Present |
|  | *B. pseudomallei* strain C-240-05-2-07 | Present | Present |
|  | *B. pseudomallei* strain C-240-05-2-08 | Present | Present |
|  | *B. pseudomallei* strain C-240-05-2-09 | Present | Present |
|  | *B. pseudomallei* strain C-274-02-1-02 | Present | Present |
|  | *B. pseudomallei* strain C-240-05-2-10 | Present | Present |
|  | *B. pseudomallei* strain C-274-02-1-03 | Present | Present |
|  | *B. pseudomallei* strain C-274-02-1-01 | Present | Present |
|  | *B. pseudomallei* strain C-274-02-1-04 | Present | Present |
|  | *B. pseudomallei* strain C-274-02-1-05 | Present | Present |
|  | *B. pseudomallei* strain C-274-02-1-07 | Present | Present |
|  | *B. pseudomallei* strain C-274-02-1-06 | Present | Present |
|  | *B. pseudomallei* strain C-274-02-1-08 | Present | Present |
|  | *B. pseudomallei* strain C-274-02-1-09 | Present | Present |
|  | *B. pseudomallei* strain C-278-05-1-01 | Present | Present |
|  | *B. pseudomallei* strain C-274-02-1-10 | Present | Present |
|  | *B. pseudomallei* strain C-278-05-1-02 | Present | Present |
|  | *B. pseudomallei* strain C-278-05-1-03 | Present | Present |
|  | *B. pseudomallei* strain C-278-05-1-04 | Present | Present |
|  | *B. pseudomallei* strain C-278-05-1-07 | Present | Present |
|  | *B. pseudomallei* strain C-278-05-1-06 | Present | Present |
|  | *B. pseudomallei* strain C-278-05-1-05 | Present | Present |
|  | *B. pseudomallei* strain C-278-05-2-09 | Present | Present |
|  | *B. pseudomallei* strain C-278-05-2-08 | Present | Present |
|  | *B. pseudomallei* strain C-320-05-2-02 | Present | Present |
|  | *B. pseudomallei* strain C-278-05-2-10 | Present | Present |
|  | *B. pseudomallei* strain C-320-05-2-03 | Present | Present |
|  | *B. pseudomallei* strain C-320-05-2-06 | Present | Present |
|  | *B. pseudomallei* strain C-320-05-2-04 | Present | Present |
|  | *B. pseudomallei* strain C-320-05-2-05 | Present | Present |
|  | *B. pseudomallei* strain C-320-05-2-10 | Present | Present |
|  | *B. pseudomallei* strain C-320-05-2-09 | Present | Present |
|  | *B. pseudomallei* strain C-320-05-2-08 | Present | Present |
|  | *B. pseudomallei* strain C-328-05-1-04 | Present | Present |
|  | *B. pseudomallei* strain C-328-05-1-03 | Present | Present |
|  | *B. pseudomallei* strain C-328-05-1-01 | Present | Present |
|  | *B. pseudomallei* strain C-328-05-1-02 | Present | Present |
|  | *B. pseudomallei* strain C-328-05-1-05 | Present | Present |
|  | *B. pseudomallei* strain C-328-05-1-06 | Present | Present |
|  | *B. pseudomallei* strain C-328-05-1-07 | Present | Present |
|  | *B. pseudomallei* strain C-328-05-1-08 | Present | Present |
|  | *B. pseudomallei* strain C-328-05-1-09 | Present | Present |
|  | *B. pseudomallei* strain C-328-05-1-10 | Present | Present |
|  | *B. pseudomallei* strain TX2018b | Present | Present |
|  | *B. pseudomallei* strain AZ1999 | Present | Present |
|  | *B. pseudomallei* strain 3001546678 | Present | Present |
|  | *B. pseudomallei* strain MSHR0643 | Present | Present |
|  | *B. pseudomallei* strain Bp9801 | Present | Present |
|  | *B. pseudomallei* strain S-553 | Present | Present |
|  | *B. pseudomallei* strain NA67 | Present | Present |
|  | *B. pseudomallei* strain V1514 | Present | Present |
|  | *B. pseudomallei* strain V1502 | Present | Present |
|  | *B. pseudomallei* strain V1610 | Present | Present |
|  | *B. pseudomallei* strain V1513 | Present | Present |
|  | *B. pseudomallei* strain V1607 | Present | Present |
|  | *B. pseudomallei* strain 4702 | Present | Present |
|  | *B. pseudomallei* strain 4822 | Present | Present |
|  | *B. pseudomallei* strain MSHR7406 | Present | Present |
|  | *B. pseudomallei* strain MSHR4259 | Present | Present |
|  | *B. pseudomallei* strain MSHR4117 | Present | Present |
|  | *B. pseudomallei* strain MSHR4102 | Present | Present |
|  | *B. pseudomallei* strain MSHR3946 | Present | Present |
|  | *B. pseudomallei* strain MSHR9187 | Present | Present |
|  | *B. pseudomallei* strain MSHR9170 | Present | Present |
|  | *B. pseudomallei* strain MSHR6652 | Present | Present |
|  | *B. pseudomallei* strain MSHR6542 | Present | Present |
|  | *B. pseudomallei* strain MSHR5930 | Present | Present |
|  | *B. pseudomallei* strain MSHR5299 MSHR5922 | Present | Present |
|  | *B. pseudomallei* strain 134 | Present | Present |
|  | *B. pseudomallei* strain 134K | Present | Present |
